# CARMA3 Promotes Colorectal Cancer Cell Motility and Cancer Stemness via YAP-Mediated NF-κB Activation

**DOI:** 10.3390/cancers13235946

**Published:** 2021-11-26

**Authors:** Ting-Yu Chang, Cheng-Tien Wu, Meei-Ling Sheu, Rong-Sen Yang, Shing-Hwa Liu

**Affiliations:** 1 Institute of Toxicology, College of Medicine, National Taiwan University, Taipei 10051, Taiwan; d03447003@ntu.edu.tw; 2 Department of Nutrition, China Medical University, Taichung 406040, Taiwan; ct-wu@mail.cmu.edu.tw; 3 Master Program for Food and Drug Safety, China Medical University, Taichung 406040, Taiwan; 4 Institute of Biomedical Sciences, National Chung Hsing University, Taichung 40227, Taiwan; mlsheu@nchu.edu.tw; 5 Department of Medical Research, Taichung Veterans General Hospital, Taichung 40705, Taiwan; 6 Department of Orthopedics, National Taiwan University Hospital, Taipei 10051, Taiwan; 7 Department of Medical Research, China Medical University Hospital, China Medical University, Taichung 406040, Taiwan; 8 Department of Pediatrics, College of Medicine, National Taiwan University & Hospital, Taipei 10051, Taiwan

**Keywords:** CARMA3, YAP, NF-κB, metastasis, cancer stem cell, colorectal cancer

## Abstract

**Simple Summary:**

CARMA3 is overexpressed in most cancers, and its expression is positively associated with poor prognosis. In this study, we evaluated the detailed mechanisms of CARMA3-mediated CRC metastasis. We found that overexpression of CARMA3 induced the expression of YAP and NF-κB activation, then elicited EMT induction to enhance cell migration and invasion. We demonstrate for the first time that YAP is a critical downstream regulator of CARMA3 in CRC. Our findings reveal a regulation axis between CARMA3 and Hippo oncoprotein YAP and further support the potential role of CARMA3 in the metastasis and cancer stemness of CRC.

**Abstract:**

CARD-recruited membrane-associated protein 3 (CARMA3) is overexpressed in various cancers and is associated with cancer cell proliferation, metastasis, and tumor progression; however, the underlying mechanisms of CARMA3 in colorectal cancer (CRC) metastasis remain unclear. Here, we found that higher CARMA3 expression was correlated with poor overall survival and metastasis in CRC patients from the TNMplot database and Human Tissue Microarray staining. Elevating CARMA3 expression promoted cell proliferation, epithelial-mesenchymal transition (EMT) induction, migration/invasion abilities, sphere formation, and cancer stem cell markers expression. Knockdown of CARMA3 decreased these processes via the EMT-related transcription factor Slug. Moreover, CARMA3 depletion significantly reduced tumor growth in mice that were consistent with the in vitro results. CRC migration/invasion could be regulated by CARMA3/YAP/Slug signaling axis using genetic inhibition of Yes-associated protein (YAP). Interestingly, CARMA3 induced activation of nuclear factor (NF)-κB through YAP expression, contributing to upregulation of Slug. YAP expression positively correlated with CARMA3, NF-κB, and Slug gene expression and poor clinical outcomes in CRC patients. Our findings demonstrate for the first time that CARMA3 plays an important role in CRC progression, which may serve as a potential diagnostic biomarker and candidate therapeutic target for CRC treatment.

## 1. Introduction

Colorectal cancer (CRC) is the third commonly diagnosed cancer and the third leading cause of cancer-related deaths worldwide in 2020 [1]. Approximately 20% of CRC patients are diagnosed with distant metastasis and the 5-year relative survival outcomes remain poor, with less than 20% [2]. It is essential to investigate genes and mechanisms involved in tumor metastasis, which can supply novel diagnostic biomarkers or therapeutic targets for CRC. CARD recruited membrane-associated protein 3 (CARMA3) belongs to the CARMA family, which includes CARMA1, CARMA2, and CARMA3. CARMA3 has been shown to be overexpressed in various human cancers [3]. Several studies reported that inhibition of CARMA3 decreased cell proliferation, migration, and tumor growth in NSCLC, bladder cancer, hepatocellular carcinoma, renal cell carcinoma, and colorectal cancer [4,5,6,7,8]. CARAM3 enhanced nuclear factor κ-light-chain-enhancer of activated B cells (NF-κB) activation by regulating the downstream IKK complex activity in response to RTKs and PKC stimulation [9,10]. Previous studies have also found that NF-κB activation drives cancer cell proliferation, metastasis, and angiogenesis in different cellular contexts [11,12]. However, the other possible molecular mechanisms of CARMA3 in CRC were largely unclear.

The Hippo pathway is a highly conserved pathway that controls organ growth through the regulation of cell proliferation, differentiation, and apoptosis [13]. Nevertheless, dysregulation of the Hippo pathway is frequently found in multiple human cancers [14]. Yes-associated protein (YAP) is a downstream transcription activator in the Hippo pathway, which is also an oncoprotein for inducing cancer initiation, progression, chemoresistance, and differentiation of cancer stem cells [15,16,17,18,19]. Overexpression of YAP has been found to be associated with poor outcomes and cetuximab resistance in CRC patients [20]. However, there were no studies exploring the relationship between CARMA3 and YAP.

In the present study, we evaluated the detailed mechanisms of CARMA3-mediated CRC metastasis. We demonstrated a novel mechanism by which CARMA3 induced YAP expression and subsequently enhanced NF-κB activation, EMT-related transcription factor Slug expression, and metastasis. We further found that YAP expression was positively correlated with CARMA3 expression and survival prognosis in patients with CRC. These findings suggest that CARMA3 may serve as a potential diagnostic marker for CRC.

## 2. Materials and Methods

### 2.1. Cell Lines

SW480, SW620, and HT-29 cells were purchased from American Type Culture Collection (ATCC, Manassas, VA, USA). HCT116 cell were obtained from the Bioresource Collection and Research Center (BCRC, Hsinchu, Taiwan). These cells were free of Mycoplasma contamination and were authenticated using STR profiling. SW480 and SW620 cells were grown in DMEM/F12 (Gibco, Thermo Fisher Scientific, Waltham, MA, USA). HT-29 cells were grown in DMEM. HCT116 cells were cultured with McCoy’s 5A medium (Sigma-Aldrich, St. Louis, MO, USA). Culture media were supplemented with 10% FBS, 100 units/mL penicillin, 100 μg/mL streptomycin, and 25 µg/mL of Amphotericin B (Sigma-Aldrich, St. Louis, MO, USA).

### 2.2. Reagents and Antibodies

Verteporfin (cat. no. SML0534) was obtained from Sigma-Aldrich (St. Louis, MO, USA). Both BAY 11-7082 (cat. no. 10010266) and TNF-α (cat. no. 32020) were purchased from Cayman Chemical (Ann Arbor, MI, USA). Protease inhibitor cocktail was purchased from Thermo Fisher Scientific (cat. no. 78430, Thermo Fisher Scientific Inc., Waltham, MA, USA). The primary antibodies for CARMA3 (cat. no. ab137383; Abcam, Cambridge, UK); E-cadherin (cat. no. sc-7870; Santa Cruz Biotechnology, Inc., Dallas, TX, USA); N-cadherin (cat. no. 14215; Cell Signaling Technology, Danvers, MA, USA); Fibronectin (cat. no. 610077; BD Transduction Laboratories); Snail (cat. no. 3879; Cell Signaling Technology, Danvers, MA, USA); Slug (cat. no. 9585; Cell Signaling Technology, Danvers, MA, USA); YAP (cat. no. 14074; Cell Signaling Technology, Danvers, MA, USA) and α-Tubulin (cat. no. T-5168; Sigma-Aldrich, St. Louis, MO, USA) were used. All secondary antibodies were purchased from Cell Signaling Technology (Danvers, MA, USA).

### 2.3. Western Blotting

Cells were washed with PBS, lysed in RIPA lysis buffer (20 mM Tris-base, 150 mM NaCl, 1 mM EDTA, 1 mM EGTA, and 1% NP-40) containing protease inhibitor cocktail (Thermo Fisher Scientific), incubated at −20 °C overnight, and then centrifuged at 13,000 rpm for 30 min. An equal quantity of protein from cell lysates was re-suspended in gel sample buffer, resolved by SDS-polyacrylamide gel electrophoresis, and transferred to PVDF membranes (Merck Millipore, Billerica, MA, USA). After blocking, blots were incubated with specific primary antibodies, after washing and incubating with secondary antibodies, immunoreactive proteins were visualized using an enhanced chemiluminescence detection system (Bio-Rad, Hercules, CA, USA).

### 2.4. Cell Proliferation by MTT Assay

Cells (3 × 10^3^ cells/well) were seeded into a 96-well plate and incubated for 24, 48, 72, and 96 h. Each condition was tested in triplicate wells. At the indicated time, the MTT reagent (cat. no. M2003; Sigma-Aldrich, St. Louis, MO, USA) was added with the final concentration of 0.5 mg/mL to each well and incubated for 3 h. Then, DMSO was added to dissolve the purple crystal and measured the absorbance at 570 nm by an ELISA Reader.

### 2.5. Transwell Migration and Invasion Assay

Migration and invasion assays were performed by using transwell inserts for a 24-well plate containing 8 μm pores (Corning Costar; Lowell, MA, USA). (1) For migration assay, 8 × 10^4^ cells were seeded in the top chamber and incubated for 24 h. (2) For invasion assay, the transwell inserts were pre-coated with Matrigel matrix (Corning Costar; Lowell, MA, USA) and incubated for 30 min at 37 °C and then seeding 1 × 10^5^ cells in the top chamber and incubated for 24 h. In both assays, cells were plated in a culture medium without serum in the top chamber, and a medium supplemented with serum 10% FBS was used as a chemoattractant in the lower chamber and incubated for 24 h. Cells that did not migrate or invade through the pores were removed by a cotton swab. Cells on the lower surface of the membrane were fixed with 4% paraformaldehyde and stained with crystal violet (0.05%). The areas of a cell migrating or invading through the membrane were measured by ImageJ software using images taken by a light microscope (40×, five random fields per well).

### 2.6. Sphere Formation Assay

Cells (1000 cells/well) were resuspended in serum-free medium containing B27 supplement, 20 ng/mL EGF, and 10 ng/mL basic FGF and plated on the ultra-low attachment 24-well plates (cat. no. CLS3473, Corning Costar; Lowell, MA, USA) for 14 days. The number of spheres was counted when the diameter of spheres reached 50 μm [21].

### 2.7. Plasmid Constructs and Cell Transfection and Virus Infection Assay

Full-length human CARMA3 was provided by Prof. Peter C. Lucas [11] as kindly gifts and subcloned into the *EcoRI* site of pcDNA6 (Thermo Fisher Scientific Inc., Waltham, MA, USA). Plasmids with YAP1 (cat. no. RC231269) and SLUG (cat. no. RC202365L1) expression were purchased from Origene (Rockville, MD, USA). Transfections were carried out using PrueFection reagent (System Biosciences, Palo Alto, CA, USA) according to the manufacturer’s instructions. The lentivirus *CARD10* shRNA clones (clone ID TRCN0000300324 and TRCN0000303962), *YAP1* shRNA clones (clone ID TRCN0000107266 and TRCN0000107267), pLKO.1-emptyT control clone (clone ID TRCN0000208001), and plasmids for pMD2.G and pCMV-deltaR8.91 were purchased from the National RNAi Core Facility at Academia Sinica (Taipei, Taiwan). For lentivirus production, the envelop plasmid (pMD2.G), package plasmid (pCMV-deltaR8.91), and target genes (shRNA clones) were transfected into HEK-293T cells using PureFection reagent. Harvested medium containing lentivirus after 48 h, centrifuged at 1250 rpm for 5 min, and then collected the supernatant. For lentivirus infection, 3 × 10^5^ cells/well at 6-well plate were infected with 2 mL lentivirus containing 1 mL culture medium and 8 μg/mL polybrene (cat. no. H9268, Sigma-Aldrich, St. Louis, MO, USA) for 24 h, then cells were replaced with fresh culture medium and incubated for 48 h.

### 2.8. RNA Isolation, RT-PCR and Quantitative RT-PCR (RT-qPCR)

Total RNA was extracted from cells using TRI Reagent (Thermo Fisher Scientific) and reverse-transcribed into cDNA using M-MLV reverse transcriptase (Thermo Fisher Scientific, Waltham, MA, USA) based on the manufacturer’s instructions. RT-qPCR was performed using the StepOne system (Applied Biosystems, Waltham, MA, USA). The relative mRNA expression was normalized to the mean of the reference gene *GAPDH*.

### 2.9. Human Tissue Microarray (TMA) and Immunohistochemistry (IHC) Staining

CARMA3 was detected in the established human CRC TMA from 59 paired normal (CDN4) colon and rectum tissues and tumor (CD4) tissues, and human colorectal cancer metastasis-normal tissues (CDA3) (SuperBioChips, Seoul, Korea) by IHC staining using an IHC kit (cat. no. ab64264, Abcam, Cambridge, UK). Briefly, TMA sections were deparaffinized using a Sub-X clearing medium (Leica Biosystems Inc., Buffalo Grove, IL, USA) and rehydrated in gradient ethanol solutions. Then, antigen retrieval was used by Protease from *Streptomyces griseus* (cat. no. P5147, Sigma-Aldrich, St. Louis, MO, USA) and applied with Protein Block for 10 min. CARMA3 antibody (1:200; cat. no. ab140204, Abcam, Cambridge, UK) was utilized for staining and incubated overnight at 4 °C, followed by incubation with HRP-labeled secondary antibody. The sections were stained with DAB substrate, counterstained with hematoxylin, and mounted. The results were quantified by ImageJ software (version 1.51; National Institutes of Health, Bethesda, MD, USA) with the IHC profiler plugin as described previously [22]. It was classified into two groups based on the intensity and extent of staining: low-expression group (score 0 and 1) and high-expression group (score 2 and 3). Score 0 for the negative staining; Score 1 for the positive staining in ≤20%; Score 2 for the positive staining in 20–50% and score 3 for strong staining in >50%.

### 2.10. Animal Xenograft Experiment

Five-week-old male Nude mice were supplied by the National Laboratory Animal Center (Taipei, Taiwan) and maintained in accordance with the Guide of Association for Assessment and Accreditation of Laboratory Animal Care International. The experimental protocol was approved by the Institutional Animal Care and Use Committee of the Laboratory Animal Centre at College of Medicine, National Taiwan University (Taipei, Taiwan). Animals were acclimatized and housed under pathogen-free conditions. For the tumor growth assay, 5 × 10^6^ tumor cells were suspended in 50 μL PBS and mixed with 50 μL Matrigel (BD Biosciences, San Jose, CA, USA) and subcutaneously injected into the dorsal flanks of mice (*n* = 6/group). Tumor volumes were measured two times in a week with tumor length (*L*) and width (*W*) using calipers and calculated with the formula *LW*^2^/2.

### 2.11. Statistical Analysis

All statistical analyses were performed with Graphpad Prism software (Version 6.0; San Diego, CA, USA) and data were presented as the means ± S.D. from at least three independent experiments. The one-way analysis of variance (ANOVA) followed by the Tukey post-hoc tests was used to analyze the significant difference among groups. *p* values of less than 0.05 were considered to be statistically significant.

## 3. Results

### 3.1. CARMA3 Overexpression Correlates with Poor Prognosis of Colorectal Cancers (CRC)

**Figure 1:**
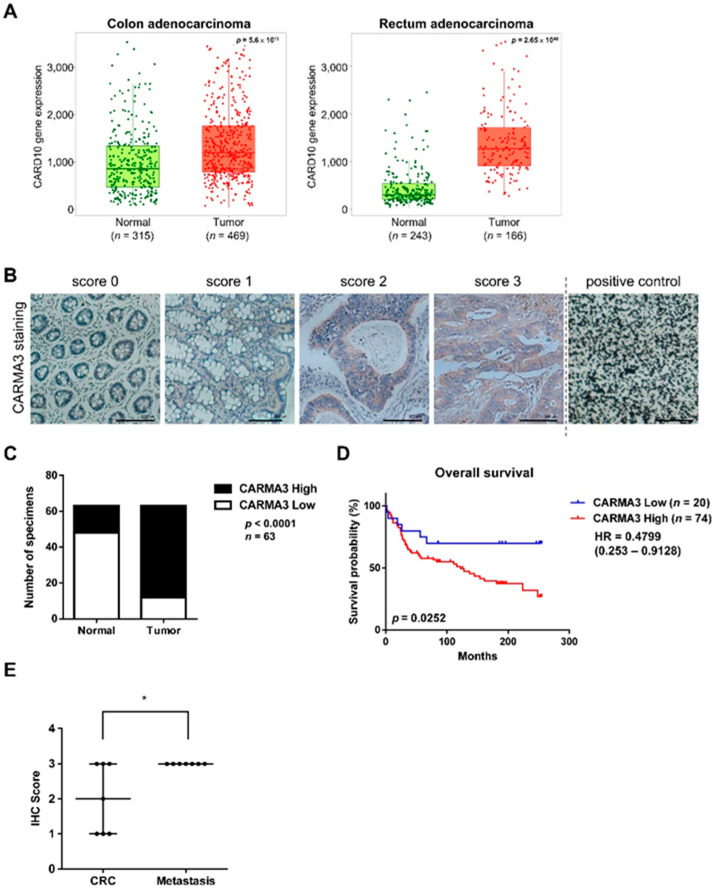
Higher expression of CARMA3 correlates with malignant progression of colorectal cancer (CRC). (**A**) Boxplot of CARMA3 gene expression in the colon (left) and rectum adenocarcinoma (right) were determined for comparing paired normal and tumor samples, which were downloaded from TNMplot. The dataset was analyzed by the Mann–Whitney U test. (**B**) CARMA3 immunohistochemical (IHC) staining in the colon and CRC TMA tissues with scores of 0–3 was shown. Carbon was used as a positive control. Magnification: 200×. Scale bar, 100 μm. (**C**) Quantification for IHC staining of CARMA3 in paired specimens of CRC and adjacent normal colon tissues was shown. The Chi-square analysis followed by Fisher’s exact test was used to analyze the significant difference. (**D**) Kaplan–Meier analysis of the overall survival of 94 colorectal cancer patients in TMA with low and high expression of CARMA3 (*p* = 0.0252, log-rank test, HR = 04799) was shown. CARMA3 expression was classified according to the median of the IHC score of specimens. (**E**) CARMA3 expression positively correlated with metastatic tissues compared to paired specimens of CRC tissues in TMA (*n* = 7). *, *p* < 0.05 by paired two-tailed Student’s *t*-tests.

To elucidate the clinical significance of CARMA3 expression in pan-cancer patients, we evaluated *CARD10* gene expression in both tumor and normal tissues of CRC cancers using the TNMplot online database, which is a web tool for the comparison of gene expression in normal, tumor and metastatic tissues [23]. As shown in Figure 1A, significantly higher expressions of *CARD10* were observed in colon and rectum tumors compared to normal tissues. In addition to the online database, we performed IHC staining of CARMA3 using CRC tissue microarray (TMA). The representative examples with different CARMA3 scores and positive staining using carbon as control were shown in Figure 1B. The IHC results also indicated that higher levels of CARMA3 expression were detected in CRC tissues than adjacent normal tissues (Figure 1C), and significantly correlated with poor survival outcomes compared to those with low levels of CARMA3 by Kaplan–Meier survival analysis (Figure 1D). Using the paired CRC and metastatic tissues in TMA, we observed that higher levels of CARMA3 scores in metastatic tissues compared to non-metastatic CRC tissues (Figure 1E). Although the fold change in CARD10 gene expression compared with tumor and normal species is not high (but statistically significant), the values from TNMplot online database of the two most important pieces of new biomarker development, sensitivity (the proportion of tumors which have higher expression than normal at a given cutoff) and specificity (the proportion of tumors divided by the total sum of all tumors and normal over the given cutoff), are high (File S1). Consistently, the expression of CARMA3 from the TMA IHC staining is significantly higher in CRC tumor tissues than adjacent normal tissues (Figure 1C). These results suggest that CARMA3 is a potential predictor for tumor progression and poor prognostic marker in CRC patients.

### 3.2. CARMA3 Expression Is Critical for CRC Aggressive Abilities

**Figure 2:**
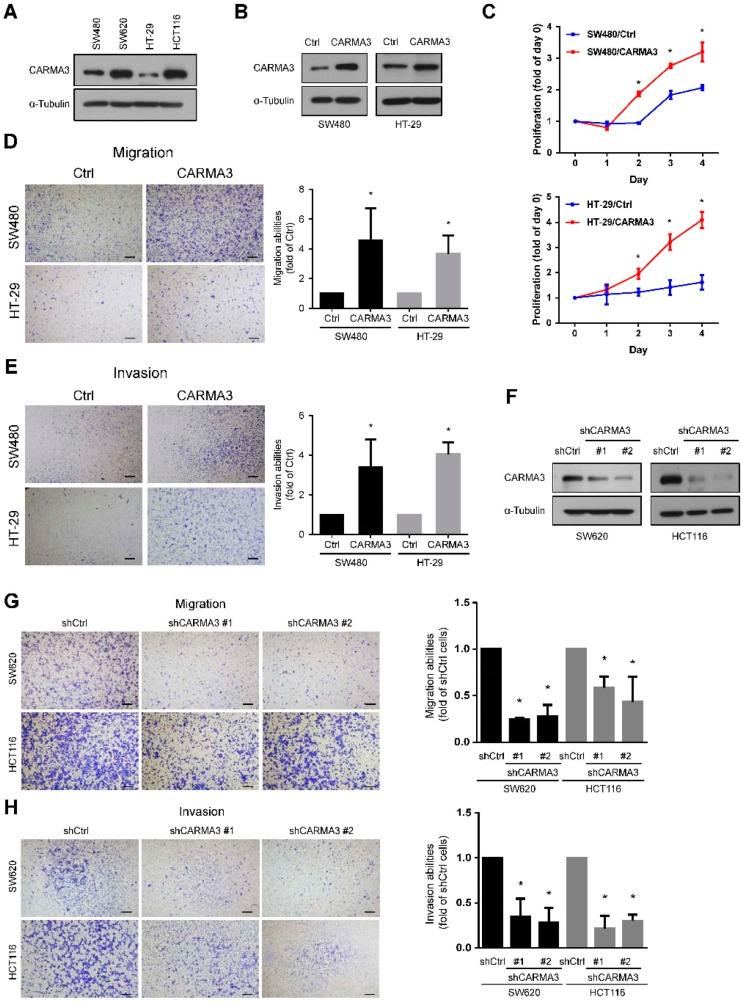
CARMA3 expression is critical for CRC’s aggressive abilities. (**A**) The basal expression level of CARMA3 in SW480, SW620, HT-29, and HCT116 cells. α-Tubulin was used as the internal protein loading control. (**B**) Overexpression efficiencies of CARMA3 were examined by Western blotting in both SW480 and HT-29 cells. Ctrl represented as control cells. (**C**) Proliferation curves were analyzed in both SW480 and HT-29 cells with CARMA3-overexpressed and control cells for four days by MTT assay. Proliferation rates were significantly higher in CARMA3-overexpressed cells compared to control cells. *, *p* < 0.05 (Student’s *t*-tests). (**D**,**E**) Both transwell migration and invasion assays of CARMA3 overexpression in SW480 and HT-29 cells were shown. Results are shown as the mean ± S.D. of three independent experiments. *, *p* < 0.05 (Student’s *t*-tests). Scale bar, 100 μm. (**F**) Knockdown efficiencies of CARMA3 by shRNAs of two clones in both SW620 and HCT116 cells were analyzed by Western blotting. (**G**,**H**) Both transwell migration and invasion abilities were determined in CARMA3-knocked down cells compared to their shRNA control (shCtrl) cells. Results are shown as the mean ± S.D. of three independent experiments. *, *p* < 0.05. Magnification: 40×. Scale bar, 100 μm.

To confirm the functional effects of CARMA3 in CRC cells, we performed the cell proliferation, migration, and invasion analysis in CRC cell lines. We have examined the basal expression of CARMA3 in SW480, SW620, HT-29, and HCT116 cells and the results were shown in Figure 2A. We found that CARMA3 was lower expressed in SW480 and HT-29 cells, higher expressed in SW620 and HCT116 cells. According to the result, we overexpressed CARMA3 in SW480 and HT-29 cells and knocked down CARMA3 in SW620 and HCT116 cells. Overexpression of CARMA3 in both SW480 and HT-29 cells (Figure 2B) significantly increased the cell proliferation abilities compared to control cells (Figure 2C). The migration and invasion abilities were analyzed by transwell migration/invasion assays of these cells. As shown in Figure 2D,E, both migration and invasion abilities were significantly increased in CARMA3-overexpressed cells. Moreover, the effects of CARMA3 depletion on the cell motility of CRC cells were also determined. The results showed that the knockdown of CARMA3 in both SW620 and HCT116 cells (Figure 2F) significantly suppressed the migration and invasion abilities (Figure 2G,H).

**Figure 3:**
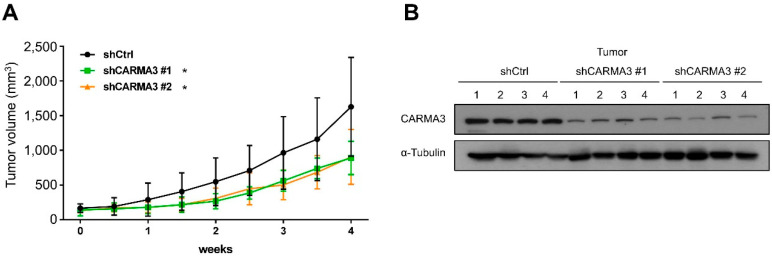
CARMA3 promotes CRC growth in a xenograft mouse model. (**A**) SW620 cells with stable expression of control or CARMA3 shRNA were subcutaneously injected into the dorsal flanks of Nude mice and measured the tumor volume two times a week for four weeks. Results are represented as the mean ± S.D. *, *p* < 0.05, vs. shCtrl groups (*n* = 6 per group). (**B**) The protein expression level of CARMA3 in xenograft tumors was analyzed by Western blot assay (full WB in File S2). α-Tubulin was used as the internal protein loading control.

To determine the effects of CARMA3 on tumor growth and progression in vivo, we subcutaneously implanted CARMA3-silenced or scrambled shRNA SW620 cells into the dorsal region of Nude mice and measured the tumor growth. The tumor volumes were significantly suppressed in CARMA3-silenced groups compared to the scrambled control groups (Figure 3A). In addition, we examined the CARMA3 protein level from tumors also showed the inhibition of CARMA3 protein expression in CARMA3-silenced groups (Figure 3B). Taken together, these results demonstrate that CARMA3 is crucial for induction of proliferation, migration, and invasion in vitro and in vivo tumor growth capability.

### 3.3. CARMA3 Expression Modulates Cancer Stemness

**Figure 4:**
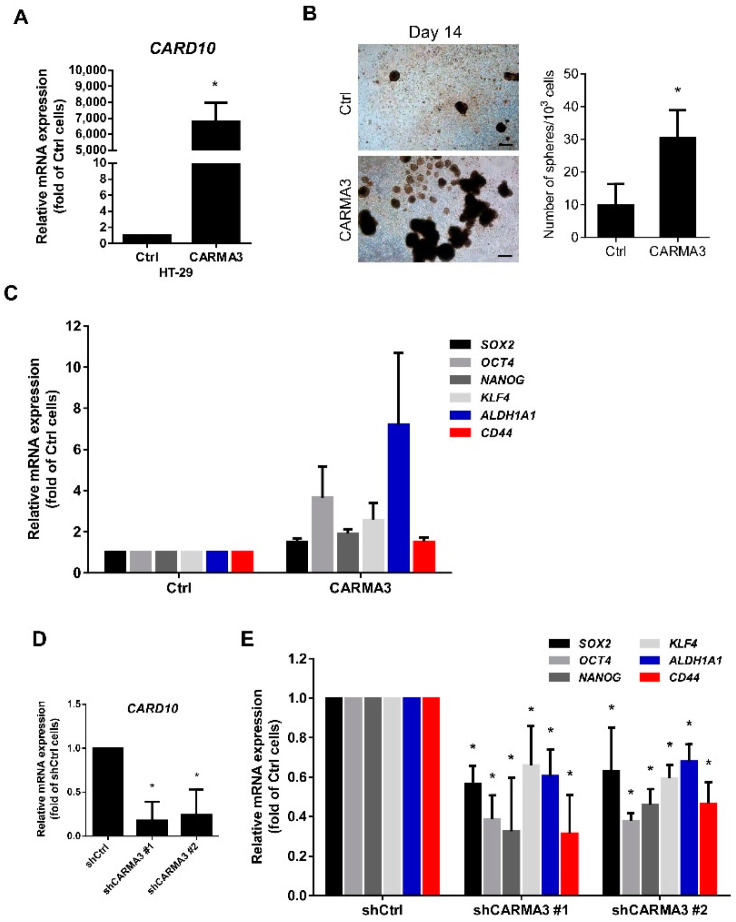
CARMA3 enhances cancer stem cell properties. (**A**) The relative mRNA expression of CARMA3 was examined in CARMA3-overexpressed and control HT-29 cells using RT-qPCR analysis. Results are shown as the mean ± S.D. of three independent experiments. *, *p* < 0.05. (**B**) The sphere formation (top) and the number of spheres (bottom) in CARMA3-overexpressed and control HT-29 cells were shown. Magnification: 40 ×. Scale bar, 100 μm. (**C**) The levels of CSC marker expression (*SOX2*, *OCT4*, *NANOG*, *KLF4*, *ALDH1A1*, and *CD44*) were measured by RT-qPCR in the indicated HT-29 cells. Results are presented as the mean ± S.D. of three independent experiments. *, *p* < 0.05. (**D**) The knockdown efficiencies of CARMA3 in SW620 cells were examined by RT-qPCR analysis. Results are presented as the mean ± S.D. of three independent experiments. *, *p* < 0.05. (**E**) The relative mRNA expression of CSC markers in CARMA3-depleted cells was measured by RT-qPCR analysis. Results are shown as the mean ± S.D. of three independent experiments. *, *p* < 0.05.

Increasing evidence suggests that cancer stem cells (CSCs) may directly or indirectly induce metastasis and tumorigenesis [24,25]. To validate the role of CARMA3 in cancer stem cell properties regulation, we performed the sphere formation assay. Ectopic expression of CARMA3 mRNA expression (Figure 4A) significantly induced the sphere formation in HT-29 cells (Figure 4B). The gene expression of CRC cancer stem cell markers, including SOX2, OCT4, NANOG, KLF4, ALDH1A1, and CD44, were all highly expressed in CARMA3-overexpressed cells compared to control cells (Figure 4C). In addition, suppression of CARMA3 mRNA expression (Figure 4D) in HCT116 cells decreased the expression of these cancer stem cell markers by qRT-PCR analysis (Figure 4E). These results suggest that CARMA3 expression has a critical role in enhancing the cancer stem cell population of CRC.

### 3.4. CARMA3 Enhanced EMT by Upregulating of Slug

**Figure 5:**
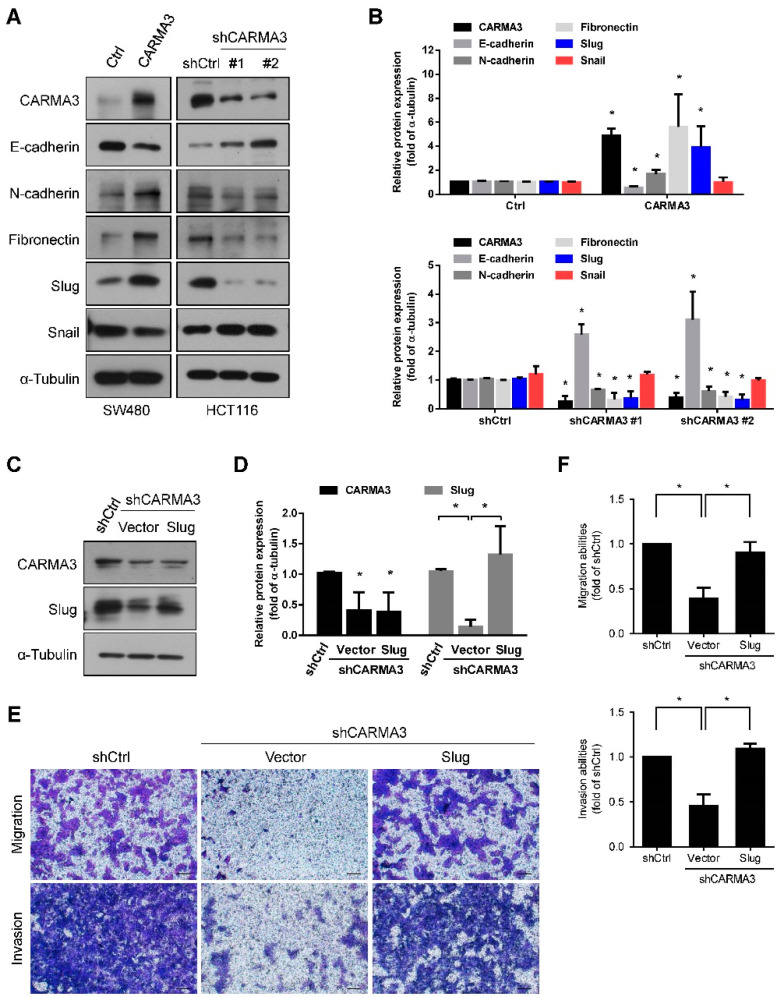
CARMA3 induced EMT by upregulation of Slug. (**A**,**B**) The protein expression levels of indicated EMT-related proteins were determined and qualified in CARMA3-overexpressed and silenced cells by the Western blot assay (full WB in File S2). Results are shown as the mean ± S.D. of three independent experiments. *, *p* < 0.05, compared to SW480/Ctrl or HCT116/shCtrl cells. (**C**,**D**) The protein expression levels of CARMA3 and Slug were analyzed by Western blot assay (full WB in File S2). α-Tubulin was used as the internal protein loading control. Each was performed in three independent experiments. Results are presented as the mean ± S.D. of three independent experiments. *, *p* < 0.05, compared to shCtrl cells or indicated cells. (**E**,**F**) The migration and invasion abilities of HCT116-knocked down CARMA3 cells with ectopic overexpression of Slug or vector control were performed in a Transwell assay. Results are shown as the mean ± S.D. of three independent experiments. *, *p* < 0.05, compared to indicated cells. Magnification: 40×. Scale bar, 100 μm.

Most evidence suggests that epithelial-mesenchymal transition (EMT) plays an important role in cancer metastasis and progression [26]. To explore the detailed mechanisms of CARMA3 in the regulation of CRC mobility, we examined the expression of EMT-related markers and transcription factors. Overexpression of CARMA3 decreased the epithelial marker E-cadherin protein expression, increased the mesenchymal markers N-cadherin and Fibronectin protein expression, and the EMT-related transcription factor, Slug, but not Snail (Figure 5A,B). Knockdown of CARMA3 in SW620 cells consistently reversed the changes in EMT-related markers and transcription factor Slug protein expression (Figure 5A,B). To further verify the role of Slug expression in CRC cells motility, re-overexpression of Slug in CARMA3-depleted cells (Figure 5C,D) restored the cell migration and invasion abilities (Figure 5E,F), indicating that Slug is involved in CARMA3-regulated CRC cell motility. These results suggest that CARMA3 promotes cell migration and invasion by inducing Slug expression in CRC cells.

### 3.5. CARMA3 Mediated CRC Metastasis through YAP/Slug Regulation

**Figure 6:**
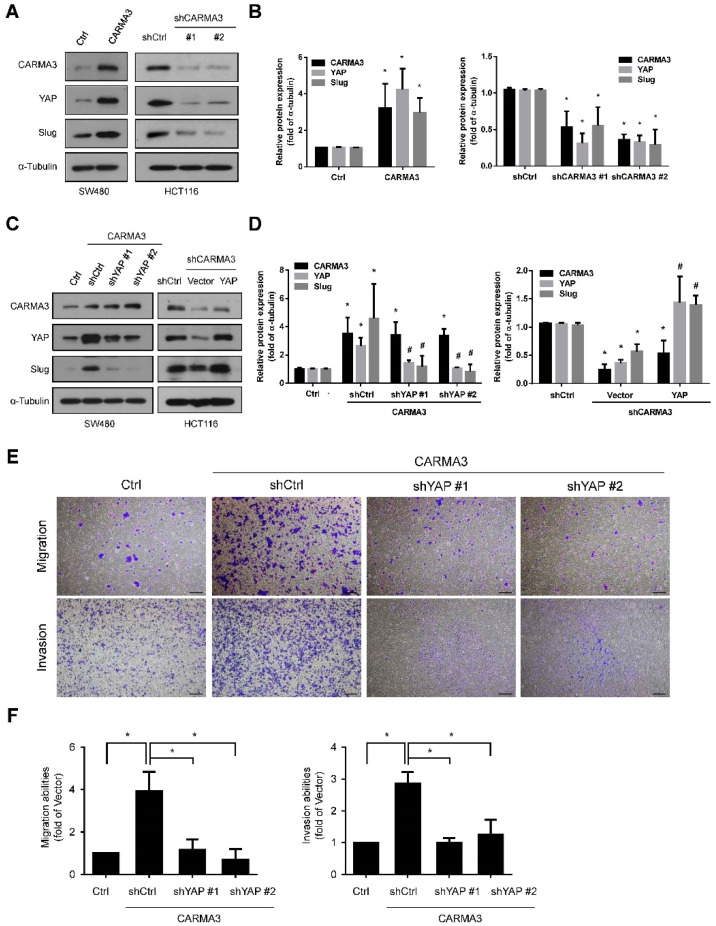
CARMA3 mediated CRC metastasis through YAP/Slug regulation. (**A**,**B**) The protein expression levels for CARMA3, YAP, and Slug in CARMA3-overexpressed or CARMA3-silenced cells were analyzed using Western blot assay (full WB in File S2). Results are presented as the mean ± S.D. of three independent experiments. *, *p* < 0.05, compared to Ctrl or shCtrl cells. (**C**,**D**) The protein expression levels of CARMA3, YAP, and Slug in cells with YAP shRNA or ectopic overexpression of YAP in the indicated cells were analyzed using Western blotting (full WB in File S2). α-Tubulin was used as the internal protein loading control. Results are presented as the mean ± S.D. of three independent experiments. *, *p* < 0.05, compared to Ctrl or shCtrl cells. #, *p* < 0.05, compared to CARMA3/shCtrl or shCARMA3/Vector cells. (**E**,**F**) The migration and invasion abilities in these indicated cells were measured by transwell migration and invasion assay. Results are shown as the mean ± S.D. of three independent experiments. *, *p* < 0.05. Magnification: 40×. Scale bar, 100 μm.

Yes-associated protein (YAP), which belongs to the Hippo pathway, is associated with cancer initiation, progression, and metastasis [27]. It has been reported that YAP promotes EMT by direct activation of Slug in CRC [28]. Therefore, we wondered whether YAP is involved in CARMA3-regulated cell metastasis. As shown in Figure 6A,B, overexpression of CARNA3 increased the protein expression of YAP and Slug, and depletion of CARMA3 reduced the protein expression of YAP and Slug, compared to control vector cells. To further ascertain the effect of YAP under CARMA3 regulation in CRC, we genetically infected shYAP lentivirus in CARMA3-overexpressed cells and transfected YAP plasmid in CARMA3-knocked down cells, and then examined the YAP and Slug protein levels. Results showed that Slug expression was decreased with YAP depletion and elevated with YAP overexpression under CARMA3 regulation, respectively (Figure 6C,D). Functionally, both migration and invasion abilities were significantly reduced in YAP-silenced cells under CARMA3 overexpression (Figure 6E,F). Taken together, these results suggest that upregulation of YAP by CARMA3 elevated Slug expression and enhanced the migration and invasion abilities in CRC cells.

### 3.6. Activation of NF-κB Is Regulated by CARMA3-Induced YAP Expression

**Figure 7:**
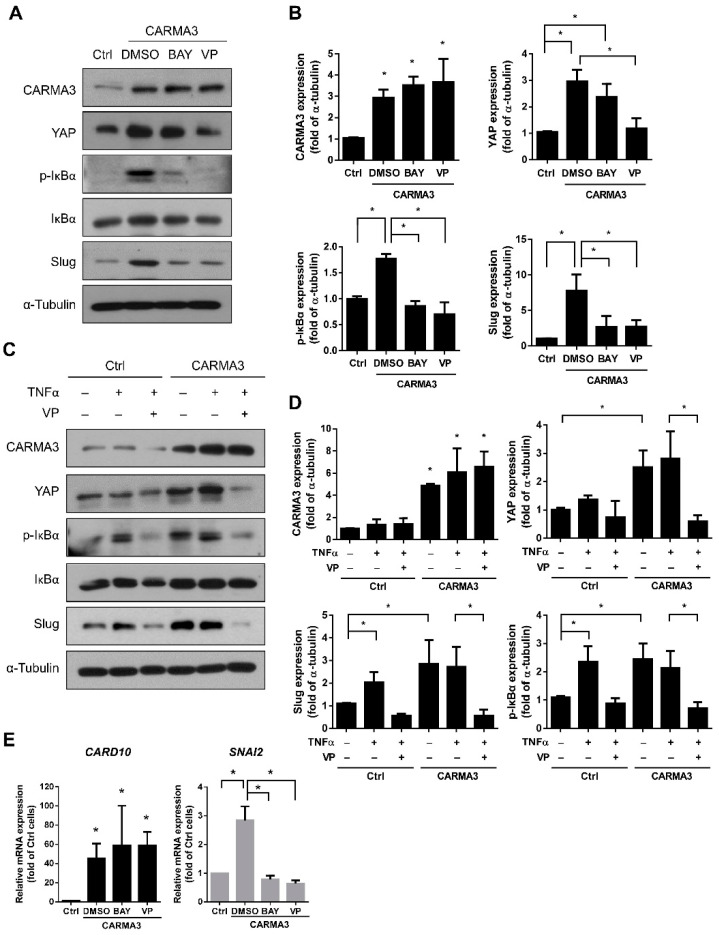
Activation of NF-κB is regulated by CARMA3-induced YAP expression. (**A**,**B**) The expression levels of CARMA3, YAP, p-IκBα, IκBα, and Slug with BAY11-7082 (BAY, 5 μM) or verteporfin (VP, 1.25 μM) treatment in CARMA3-overexpressed cells were determined by Western blot assay (full WB in File S2). Each was performed in three independent experiments. Results indicate the mean ± S.D. *, *p* < 0.05 (**C**,**D**) CARMA3-overexpressed SW480 cells were treated with TNFα (20 ng/mL) in the presence or absence of VP for 3 h and analyzed the indicated protein expression using Western blot assay (full WB in File S2). α-Tubulin was used as the internal protein loading control. Each was performed in three independent experiments. The columns indicate the mean ± S.D. *, *p* < 0.05. (**E**) The mRNA expression of *CARD10* and *SNAI2* with BAY11-7082 (BAY, 5 μM) or verteporfin (VP, 1.25 μM) treatment in CARMA3-overexpressed cells were detected by qRT-PCR. Results indicate the mean ± S.D. *, *p* < 0.05.

The role of NF-κB in inflammation, cancer initiation, and progression has been reported that NF-κB activation can be regulated by CARMA3 [3]. Therefore, we were curious about whether the NF-κB pathway was involved in the CARMA3-mediated expression of YAP. First, we examined the effects of both verteporfin (VP, YAP inhibitor) and BAY11-7082 (NF-κB inhibitor) treatment on YAP expression and NF-κB activation in CARMA3-overexpressed CRC cells. As shown in Figure 7A, the protein expression levels of YAP, phosphorylated IκBα, and Slug were inhibited by VP treatment in CARMA3-overexpressed cells compared to DMSO-treated control cells. In addition, the BAY11-7082 treatment inhibited the protein expression levels of phosphorylated IκBα and Slug, but not YAP, suggesting that NF-κB activation could be regulated by the CARMA3/YAP signaling axis (Figure 7B). In addition, the activation of NF-κB was enhanced by TNFα stimulation, which could be inhibited by YAP inhibition under CARMA3 regulation (Figure 7C,D). Moreover, the expression of Slug was also downregulated by VP treatment. Previous studies have shown that Slug mRNA expression and promoter activity were induced with TNFα treatment [29,30,31] and the Slug promoter activity was diminished by NF-κB inhibitor BAY11-7082 [32]. We also observed that Slug mRNA expression was increased by CARMA3 overexpression, and abolished with NF-κB inhibitor (BAY11-7082) and YAP inhibitor (VP) treatment (Figure 7E). These results suggest that NF-κB activation can be positively regulated by CARMA3/YAP axis, which further transcriptionally regulates Slug expression in CRC cells.

**Figure 8:**
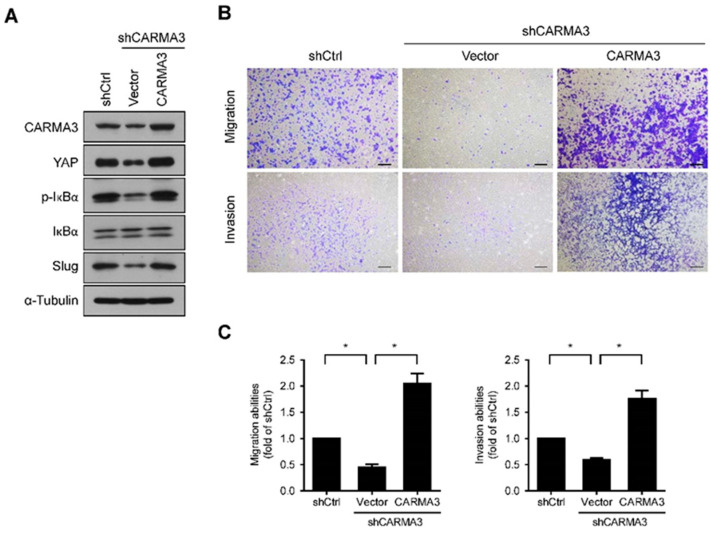
Re-overexpression of CARMA3 in CARMA3-silenced cells restores the downstream effects. (**A**) The protein expression levels of CARMA3, YAP, p-IκBα, IκBα, and Slug were determined with re-overexpression of CARMA3 in CARMA3-silenced cells by Western blot assay (full WB in File S2). α-Tubulin was used as the internal protein loading control. Each was performed in three independent experiments. (**B**,**C**) The migration and invasion abilities of HCT116-knocked down CARMA3 cells with re-overexpression of CARMA3 or vector control were performed in a transwell assay. Results are shown as the mean ± S.D. of three independent experiments. *, *p* < 0.05, compared to indicated cells. Magnification: 40×. Scale bar, 100 μm.

To further confirm the role and regulatory mechanism of CARMA3 in CRC invasiveness, we restored the expression of CARMA3 in CARMA3-knocked down cells and determined the downstream effects. Overexpression of CARMA3 in HCT116/shCARMA3 cells significantly abolished the inhibition of YAP, NF-kB, and Slug expression (Figure 8A). Moreover, the migration and invasion abilities were restored in re-overexpressed CARMA3 cells (Figure 8B,C), indicating that the expression of CARMA3 is critical for CRC invasiveness.

### 3.7. CARMA3/YAP/NF-κB/Slug Signaling Pathway Is Associated with Poor Prognosis for Human Colorectal Cancer

**Figure 9:**
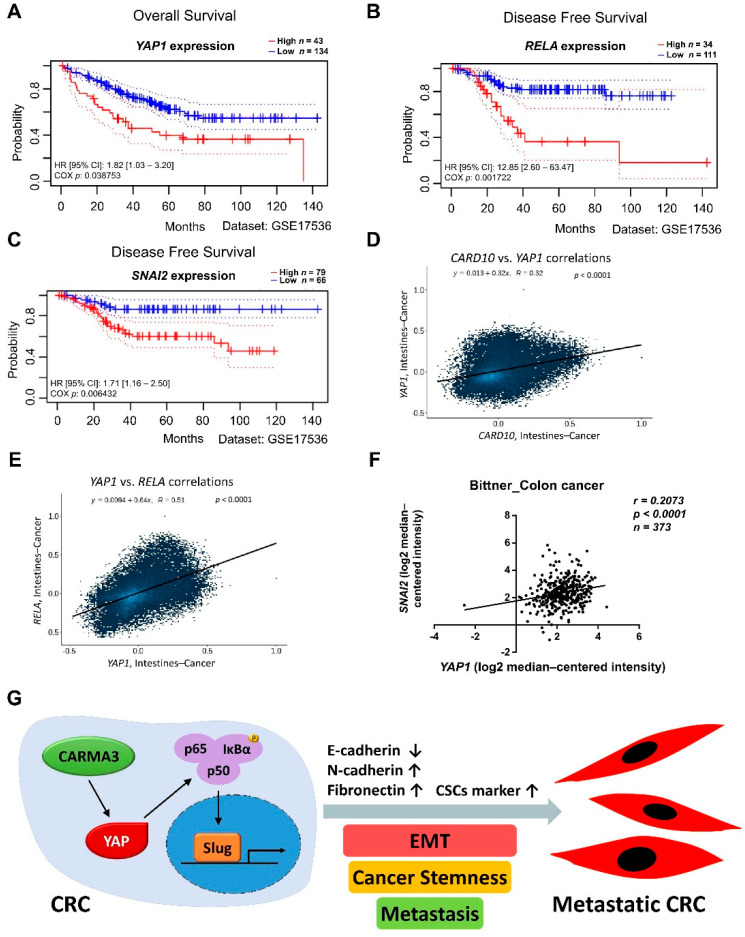
CARMA3/YAP/NF-κB/Slug signaling pathway is associated with a poor prognosis for human colorectal cancer. (**A**) Kaplan–Meier plot of overall survival of colorectal cancer patients with high and low *YAP1* expression (*p* < 0.05, log-rank test, HR = 1.82, Dataset: GSE17536). (**B**,**C**) Kaplan–Meier plot of disease-free survival of colorectal cancer patients with high and low *RELA* (*p* < 0.05, log-rank test, HR = 12.85) or *SNAI2* expression (*p* < 0.05, log-rank test, HR = 1.71, Dataset: GSE17536). (**D**,**E**) The correlation between *CARD10* and *YAP1* expression or *YAP1* and *RELA* expression in intestine cancer patients from the Correlation AnalyzeR database. Statistics from individual studies were obtained from the Correlation AnalyzeR database. The genes correlation (Pearson’s R) and *p* values were shown within each box plot. (**F**) The correlation between *YAP1* and *SNAI2* expression in colon cancer patients was analyzed from Oncomine datasets: Bittner_Colon. Statistics from individual studies were obtained from the Oncomine cancer database. The correlation coefficient (*r*), sample number (*N*), and *p* values were shown within the box plot. (**G**) A schematic model illustrating CARMA3/YAP/NF-κB/Slug pathway in CRC cells was shown. CARAM3 induces expression of YAP to activate NF-κB and induction of Slug, resulting in the enhancement of EMT, cancer stemness, and metastasis in CRC.

To clarify the clinical significance of the CARMA3/YAP/NF-κB/Slug signaling pathway in human colon cancer, we queried the PrognoScan, Correlation AnalyzeR, and Oncomine databases to analyze the survival and correlation of gene expression. High *RELA*, *YAP1*, and *SNAI2* expression levels were significantly correlated with worse survival and clinical outcomes (Figure 9A–C). We also observed that strong positive correlations for *CARD10*-*YAP1*, *YAP1*-*RELA*, and *YAP1*-*SNAI2* in patients with colon cancer (Figure 9D–F). Collectively, these results imply that CARMA3 may be a potent biomarker for the poor prognosis of colorectal cancer.

## 4. Discussion

Previous studies have identified that CARMA3 is highly expressed in CRC and associated with a worse prognosis [8,33]. Here, we uncover a novel mechanism by which CARMA3 induces the activation of NF-κB through YAP upregulation, leading to enhancing EMT and invasiveness in CRC. Furthermore, we demonstrate for the first time that YAP is a critical downstream regulator of CARMA3 in CRC. It is known that CARMA3 functions as a modulator in downstream NF-κB activation in response to receptor activation, such as epidermal growth factor (EGF) receptor and angiotensin II receptor, inducing tumor growth and progression [3,10,12]. In a previous study, CARMA3 depletion enhanced cisplatin sensitization and blocked cell cycle progression of ovarian cancer cells [34]. The CARMA-BCL10-MALT1 (CBM) complex promoted DNA damage-induced NF-κB activation to protect doxorubicin-induced cell death in both hematopoietic and non-hematopoietic cells [35]. Besides cancer development, CARMA3 is also involved in antiviral RLR signaling in pro-inflammatory responses [36]. Hence, it is necessary to discover the different biological functions and molecular mechanisms of CARMA3. Corresponding with previous studies, overexpression of CARMA3 was found in over 80% of human colorectal cancer tissues from our data of TMA staining and highly expressed in metastatic CRC tissues. These previous studies and our present findings support that CARAM3 may act not only as an oncoprotein but might also be a potential therapeutic target for cancer treatment.

Aberrant YAP expression is commonly observed in cancers, and upregulation of YAP plays a critical role in tumorigenesis, drug resistance, and tumor development of CRC [37,38,39]. Inactivation of YAP abolished the adenomas initiation in an Apc^Min^ mouse model of colon cancer [40]. Co-overexpression of YAP and TAZ has been shown to be positively correlated with poor overall survival of CRC patients [37]. YAP mediated the expression of EMA-related genes through induction of Sox2 and interaction with Oct4 to facilitate self-renewal [41]. It has also been reported that YAP promotes EMT by directly activating the expression of Slug, which inhibited E-cadherin expression in CRC cells [28]. Consistent with these previous studies, our findings found that inhibition of YAP by genetic knockdown or pharmacological inhibitor (VP) suppressed the protein expression of Slug, leading to reducing the migration and invasion abilities in CARMA3-overexpressed SW480 cells. Recently, positive feedback between YAP and NF-κB pathways was observed in CRC progression [39]. Directly targeting of *YAP1* promoter by p65 was demonstrated by chromatin immunoprecipitation (ChIP) assays with TNFα stimulation. Meanwhile, they also found that YAP activation can directly enhance NF-κB activity in colon cancer cells [34]. In this study, we found that suppression of YAP indeed reduced NF-κB activation, but inhibition of NF-κB did not reduce YAP expression under the regulation of CARMA3.

The existence of human CSCs has a critical role in tumorigenesis, metastasis, and drug resistance [42,43]. In addition, carcinoma cells enriched with properties of stem cells that have undergone an EMT were considered a key step in cancer progression [44,45]. OCT4, a cancer stem cell induction factor, induced CRC metastasis through the EMT process and served as a biomarker for defining CRC patients at high risk for liver metastases [46]. Another induction factor for pluripotent stem cells [47], KLF4 was overexpressed in spheroid cells and enhanced EMT in the CSCs-enriched population of CRC [48]. Moreover, it has been reported that CARMA3 promotes cancer stemness and metastasis through NF-κB-driven miR-182 expression and negative regulation of NME2 in non–small cell lung cancer (NSCLC) [49]. In the present study, our in vitro findings demonstrated that overexpression of CARMA3 in HT-29 cells promoted the sphere formation and induced expression of cancer stem cell markers.

Regarding the role of NF-κB in cancer development, NF-κB plays a pivotal role in CRC cell proliferation, apoptosis, drug resistance, angiogenesis, and metastasis [50]. Therefore, targeting the NF-κB pathway is thought to be a conceivable treatment strategy. Although more than 700 inhibitors of the NF-κB signaling pathway have been developed, no NF-κB inhibitor has been approved for clinical application until now due to unexpected side effects [51]. Hence, CARMA3-regulated YAP-mediated NF-κB activation may be a potential target signaling pathway for cancer treatment.

It has been reported that CARMA3 promoted lung cancer cell metastasis via the tail vein injection model [49]. The lung colonization in mice injected with A549/shCARMA3 cells was significantly decreased compared with control cells. These results support our hypothesis that CARMA3 may involve in cancer metastasis of CRCs. However, it is a limitation for our study that the direct evidence of CARMA3-induced CRC metastasis in an animal model is lacking. It may be possible in the future to analyze the in vivo metastasis.

## 5. Conclusions

In this study, we demonstrated for the first time that CARMA3 induced YAP expression and triggered NF-κB activation, leading to enhance EMT and cell metastasis. We also found that there was a clinically relevant relationship among *CARD10*, *YAP1*, *RELA*, and *SNAI2* genes in CRC. In addition, CARMA3 was highly expressed in metastatic tumor tissues of CRC patients. Our results provide a novel regulatory mechanism of CARMA3 by regulating the YAP/NF-κB/Slug signaling axis in CRC metastasis. These findings imply that CARMA3 may serve as a therapeutic target and a novel biomarker for predicting CRC metastasis.

## Data Availability

The data presented in this study are available from the corresponding author upon reasonable request.

## Supplementary Materials

The following are available online at https://www.mdpi.com/article/10.3390/cancers13235946/s1, File S1: The sensitivity/specificity plot of *CARD10* gene expression in colon cancer, File S2: Western blot raw data.
