# Supplementary material for: CARMA3 Promotes Colorectal Cancer Cell Motility and Cancer Stemness via YAP-Mediated NF-κB Activation
Source: Cancers (Basel). 2021 Nov 26;13(23):5946. doi: 10.3390/cancers13235946 (PMC8657120; doi:10.3390/cancers13235946)

Supplemental data

CARMA3 promotes colorectal cancer cell motility and cancer stemness via YAP-mediated NF-κB activation

Ting-Yu Chan, Cheng-Tien Wu, Meei-Ling Sheu, Rong-Sen Yang and Shing-Hwa Liu

Supplementary File S1

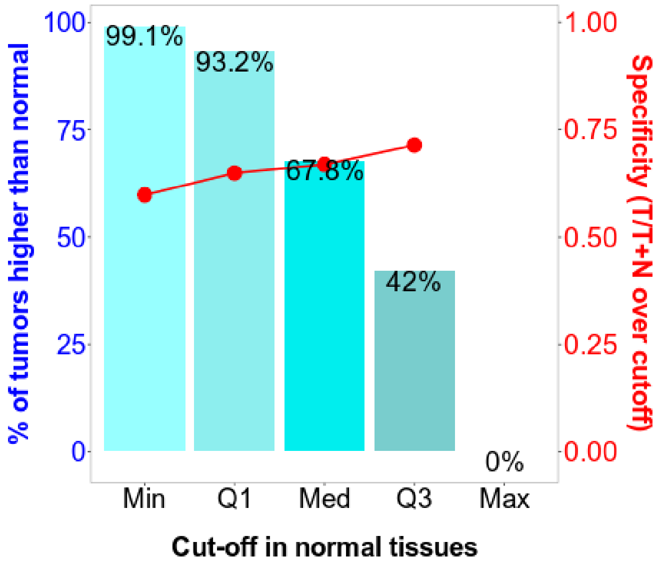

Supplementary Figure S1. The sensitivity/specificity plot of CARD10 gene expression in colon cancer when compared tumor and normal RNA-sequencing data, which were downloaded from TNMplot (TNMplot.com: A Web Tool for the Comparison of Gene Expression in Normal, Tumor and Metastatic Tissues; https://tnmplot.com/analysis/). The bars represent the sensitivity: percentage of tumors which CARD10 gene expression is higher than normal samples (blue words). The specificity is shown as red words and red line.

Supplementary File S2
Western blot raw data

1. Figure S2A

Cell: SW480, SW620, HT-29, HCT116 cells
Protein: CARMA3 (116 kDa)

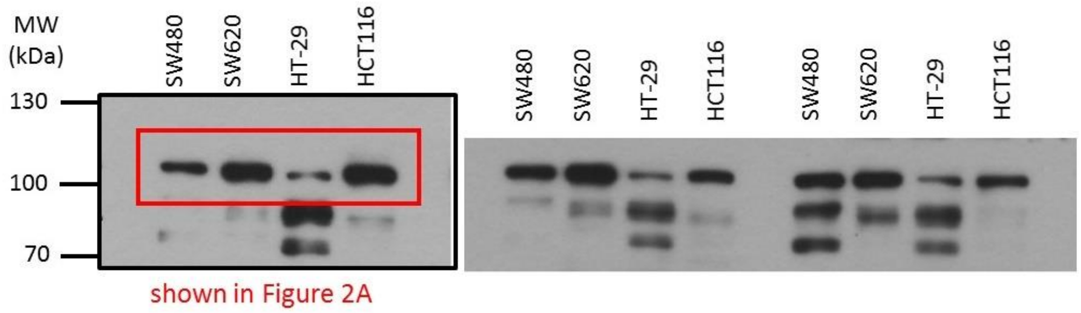

2. Figure S2A

Cell: SW480, SW620, HT-29, HCT116 cells
Protein: α-Tubilin (55 kDa)

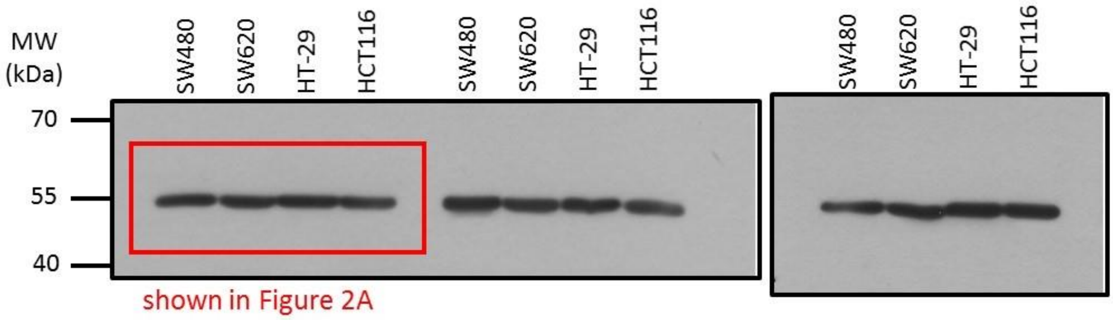

3. Figure S2B-a

Overexpression of CARMA3 by plasmid transfection
Cell: SW480 cell
Protein: CARMA3 (116 kDa)

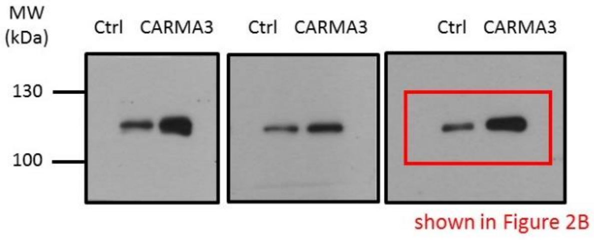

4. Figure S2B-a

Overexpression of CARMA3 by plasmid transfection
Cell: SW480 cell
Protein: α-Tubilin (55 kDa)

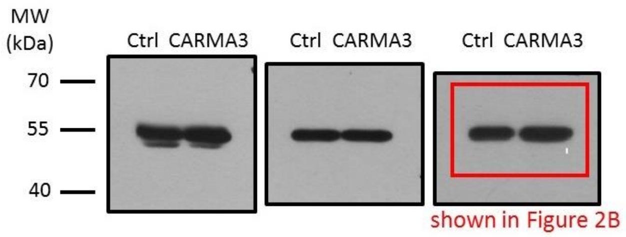

5. Figure S2B-a

Overexpression of CARMA3 by plasmid transfection
Cell: HT-29 cells
Protein: CARMA3 (116 kDa)

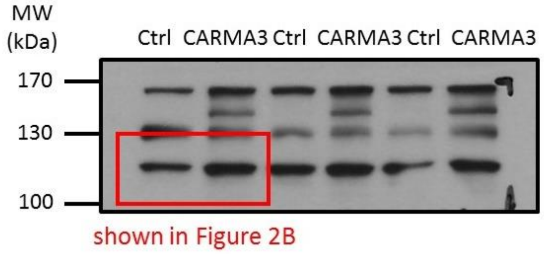

6. Figure S2B-b

Overexpression of CARMA3 by plasmid transfection
Cell: HT-29 cell
Protein: α-Tubilin (55 kDa)

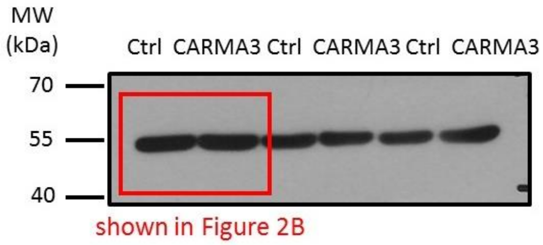

7. Figure S2F-a

Knockdown of CARMA3 by shRNA lentivirus infection
Cell: SW620 cell
Protein: CARMA3 (116 kDa)

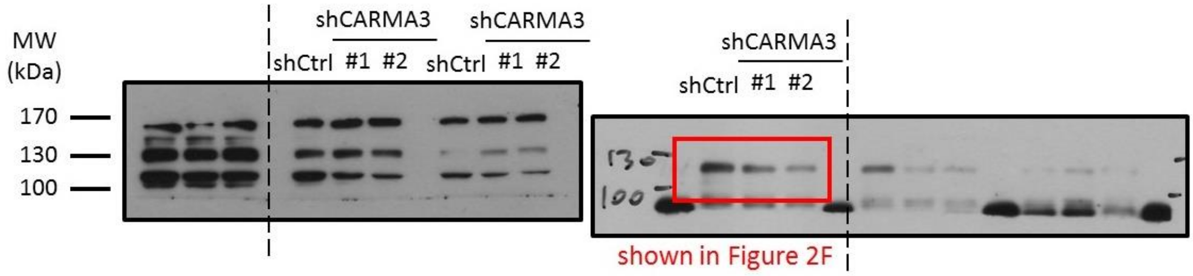

8. Figure S2F-a

Knockdown of CARMA3 by shRNA lentivirus infection
Cell: SW620 cell
Protein: α-Tubilin (55 kDa)

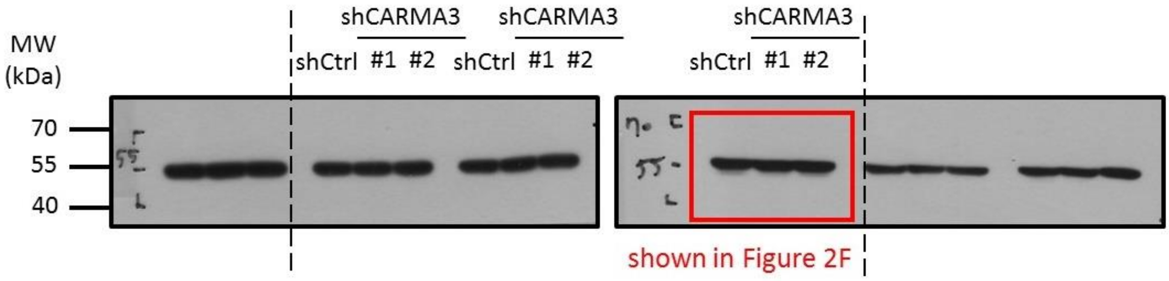

9. Figure S2F-b

Knockdown of CARMA3 by shRNA lentivirus infection
Cell: HCT116 cell
Protein: CARMA3 (116 kDa)

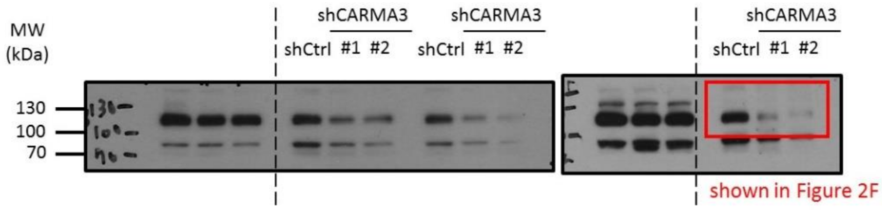

10. Figure S2F-b

Knockdown of CARMA3 by shRNA lentivirus infection
Cell: HCT116 cell
Protein: α-Tubilin (55 kDa)

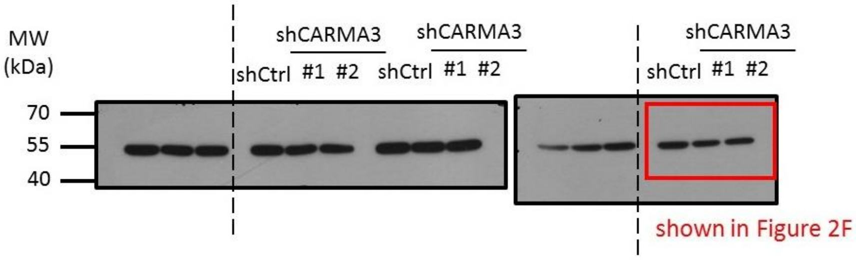

11. Figure S3B

Animal model
Knockdown of CARMA3 in HCT116 cells by shRNA lentivirus infection
Tumor tissues
Protein: CARMA3 (116 kDa)

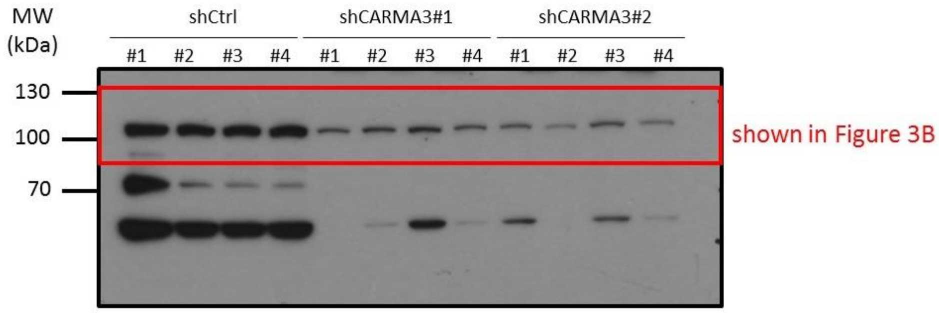

12. Figure S3B

Animal model
Knockdown of CARMA3 in HCT116 cells by shRNA lentivirus infection
Tumor tissues
Protein: α-Tubilin (55 kDa)

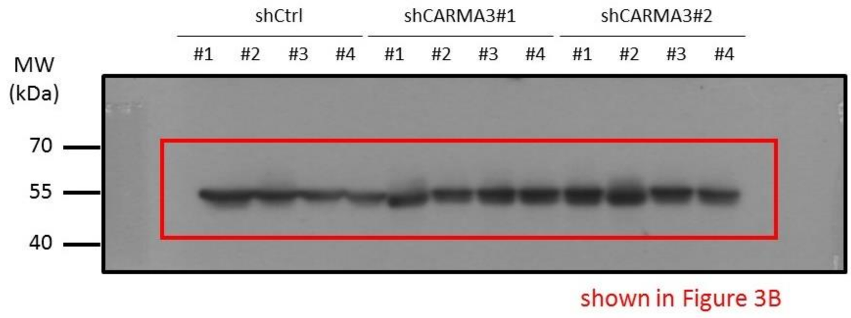

13. Figure S5A-a

Overexpression of CARMA3 by plasmid transfection
Cell: SW480 cell
Protein: CARMA3 (116 kDa)

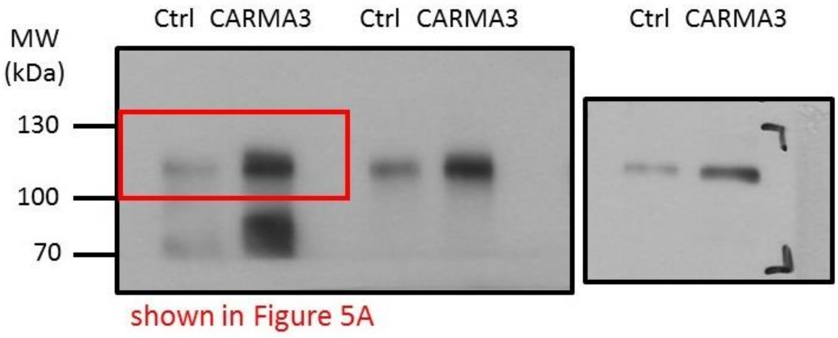

14. Figure S5A-a

Overexpression of CARMA3 by plasmid transfection
Cell: SW480 cell
Protein: E-cadherin (135 kDa)

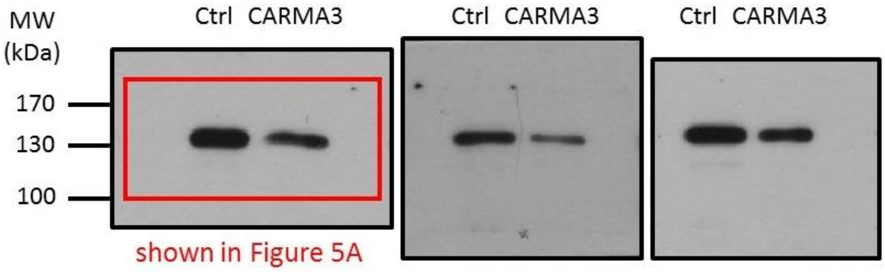

15. Figure S5A-a

Overexpression of CARMA3 by plasmid transfection
Cell: SW480 cell
Protein: N-cadherin (140 kDa)

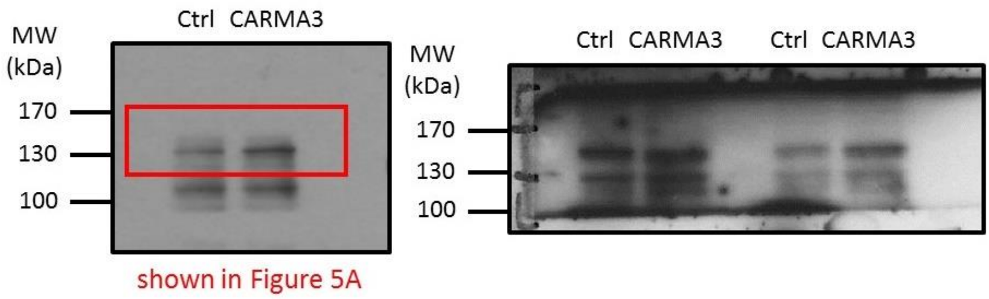

16. Figure S5A-a

Overexpression of CARMA3 by plasmid transfection
Cell: SW480 cell
Protein: Fibronectin (240 kDa)

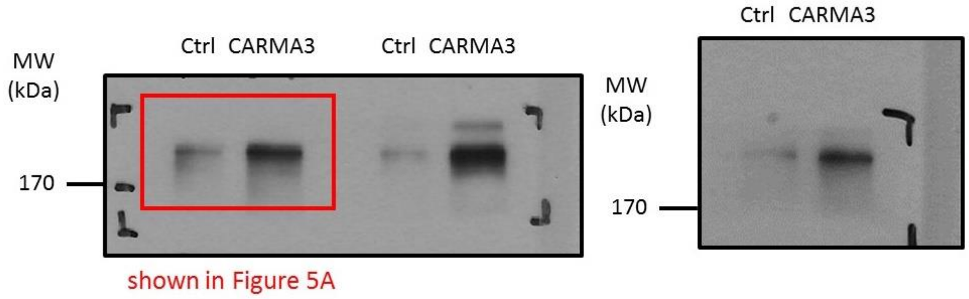

17. Figure S5A-a

Overexpression of CARMA3 by plasmid transfection
Cell: SW480 cell
Protein: Slug (30 kDa)

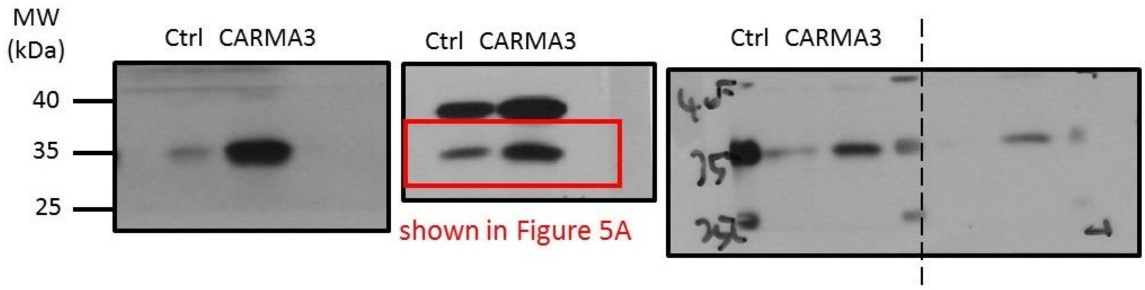

18. Figure S5A-a

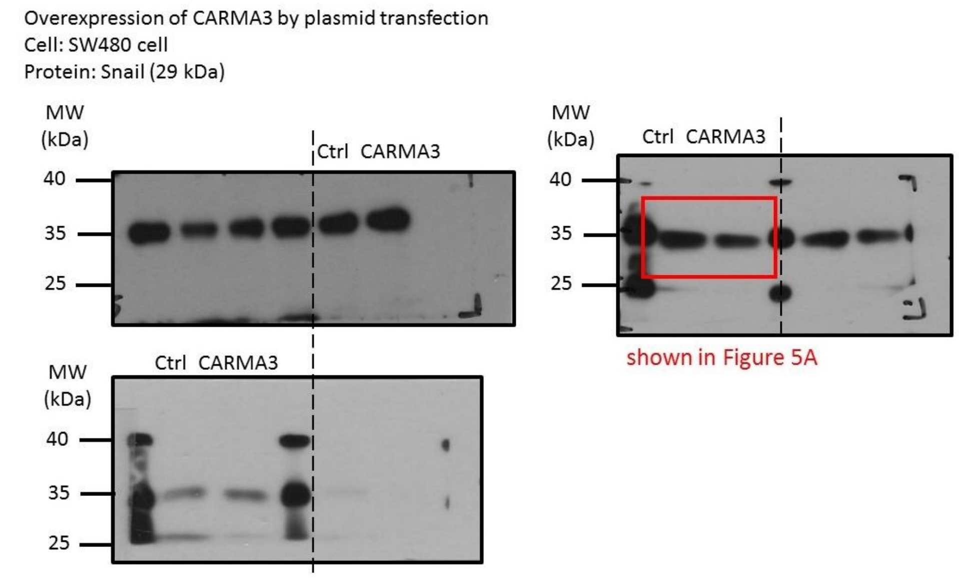

19. Figure S5A-a

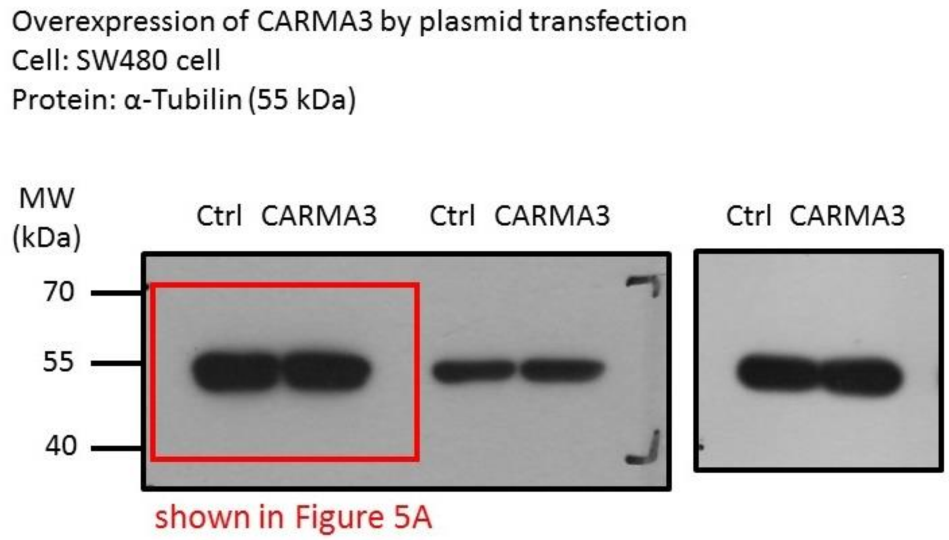

20. Figure S5A-b

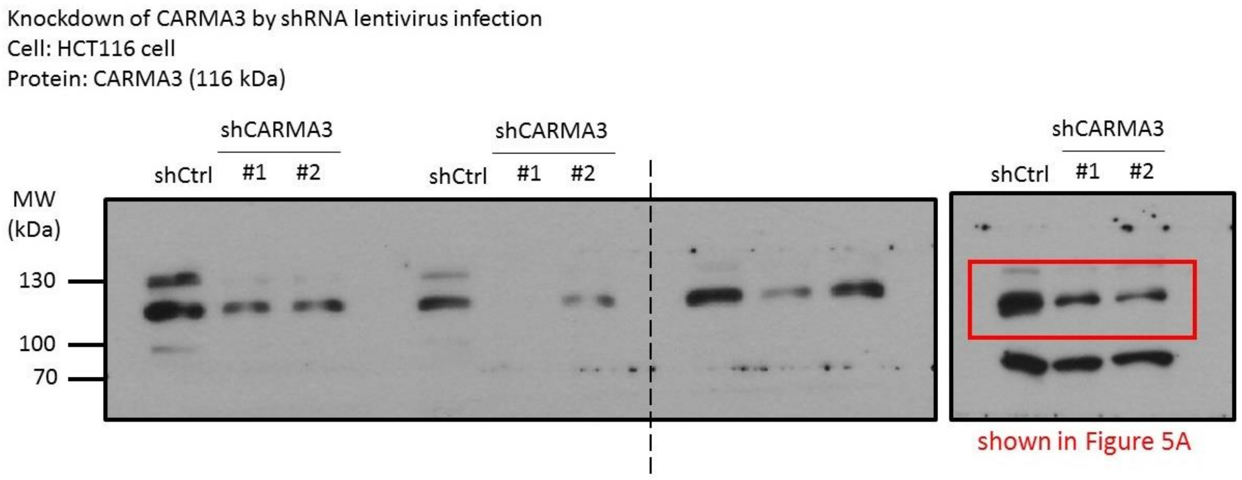

21. Figure S5A-b

Knockdown of CARMA3 by shRNA lentivirus infection
Cell: HCT116 cell
Protein: E-cadherin (135 kDa)

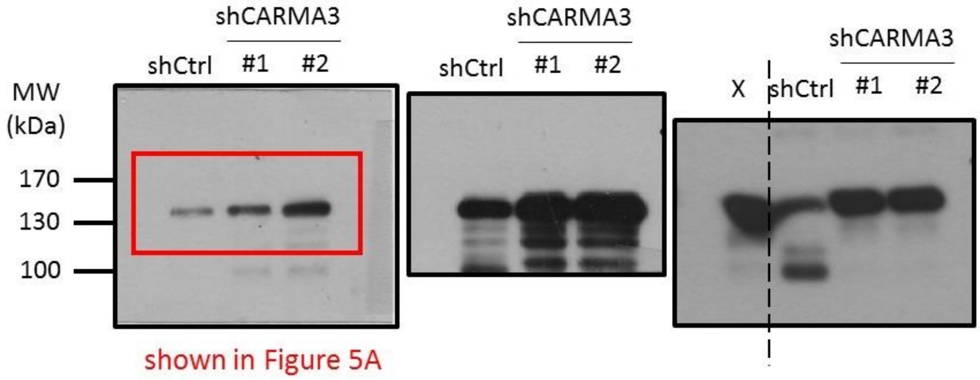

22. Figure S5A-b

Knockdown of CARMA3 by shRNA lentivirus infection
Cell: HCT116 cell
Protein: N-cadherin (140 kDa)

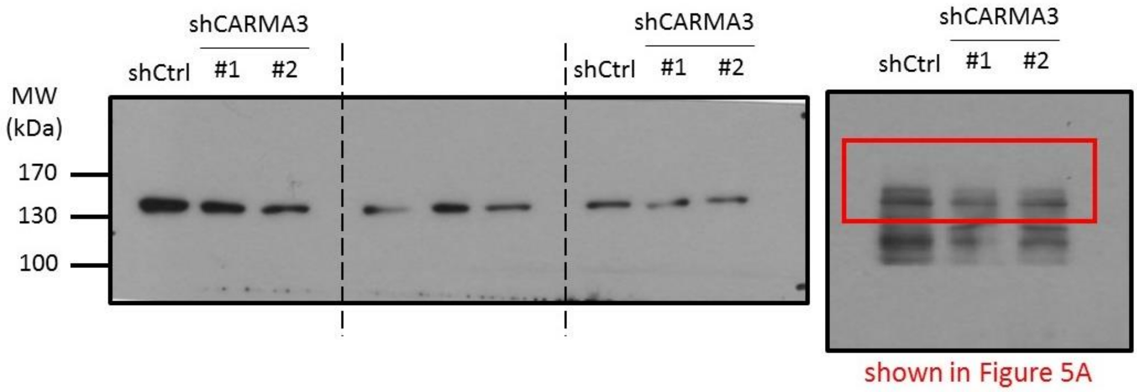

23. Figure S5A-b

Knockdown of CARMA3 by shRNA lentivirus infection
Cell: HCT116 cell
Protein: Fibronectin (240 kDa)

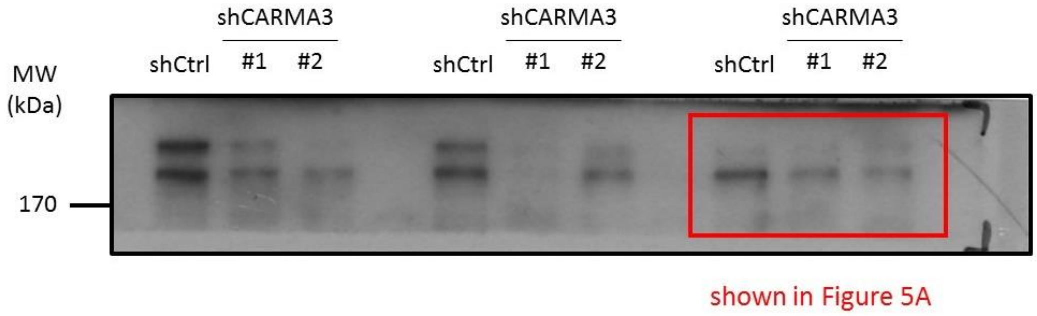

24. Figure S5A-b

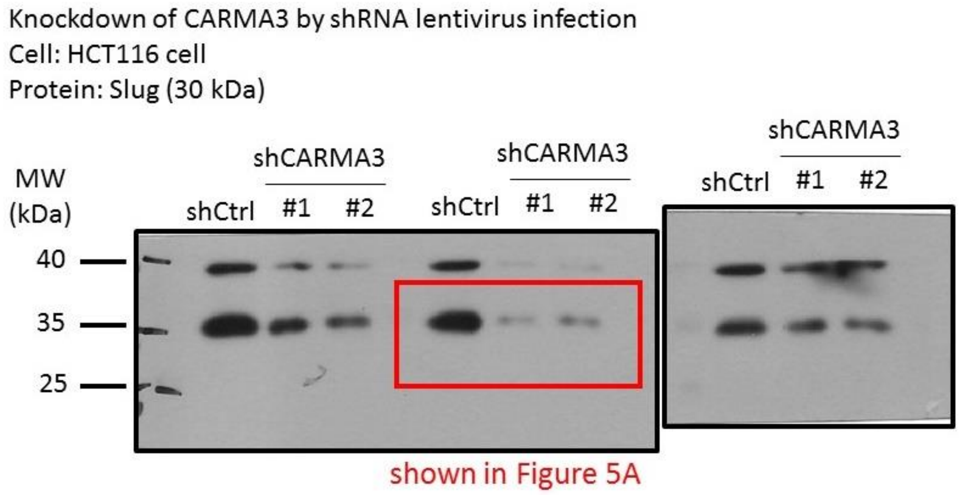

25. Figure S5A-b

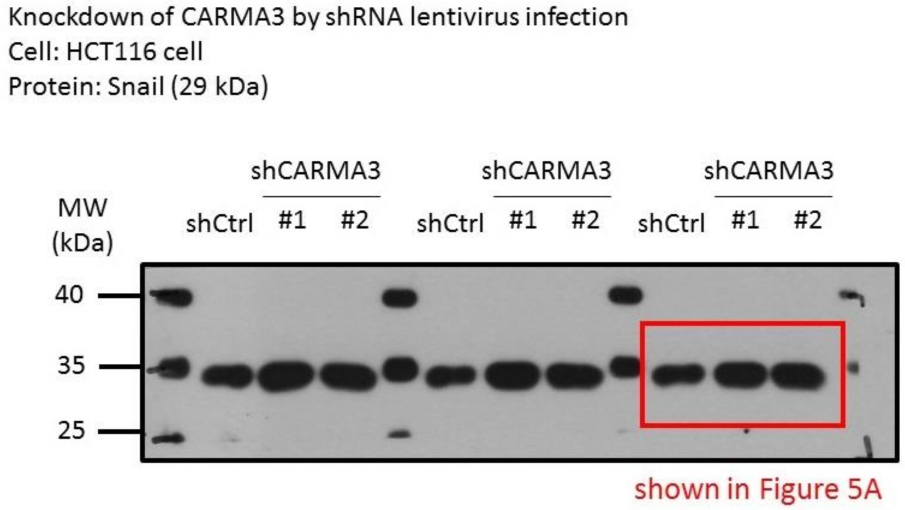

26. Figure S5A-b

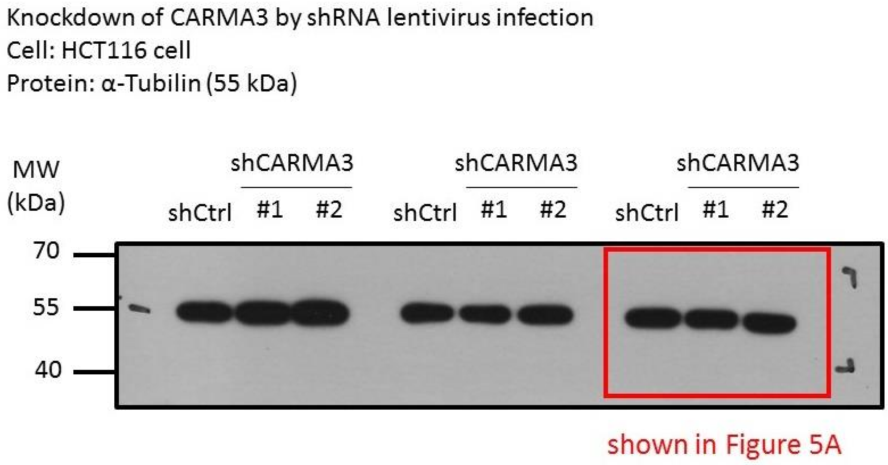

27. Figure S5C

Re-overexpression of Slug in CARMA3-knocked down cells by plasmid transfection
Cell: HCT116 cell
Protein: CARMA3 (116 kDa)

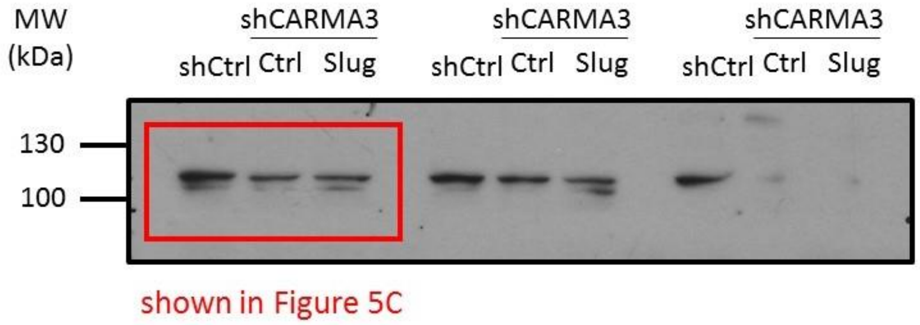

28. Figure S5C

Knockdown of CARMA3 by shRNA lentivirus infection
Cell: HCT116 cell
Protein: Slug (30 kDa)

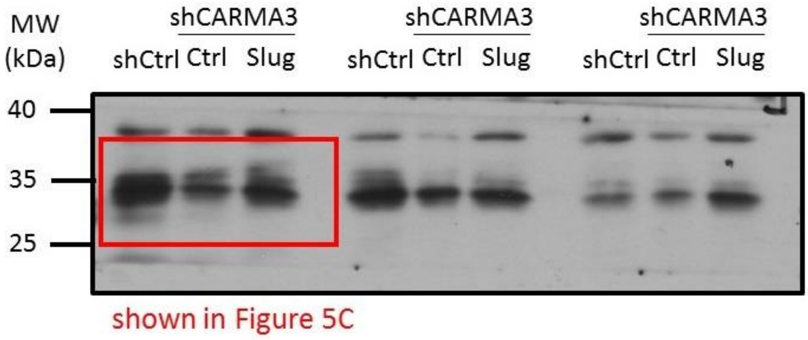

29. Figure S5C

Knockdown of CARMA3 by shRNA lentivirus infection
Cell: HCT116 cell
Protein: α-Tubilin (55 kDa)

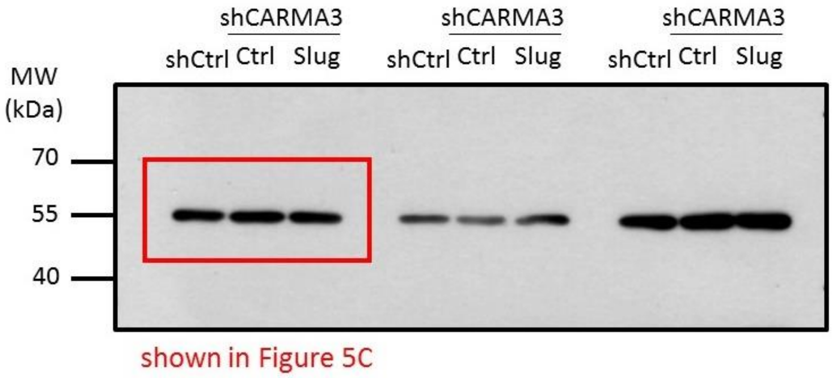

30. Figure S6A-a

Overexpression of CARMA3 by plasmid transfection
Cell: SW480 cell
Protein: CARMA3 (116 kDa)

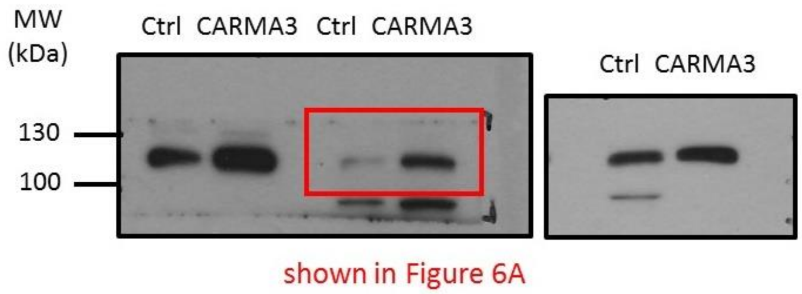

31. Figure S6A-a

Overexpression of CARMA3 by plasmid transfection
Cell: SW480 cell
Protein: YAP (65-78 kDa)

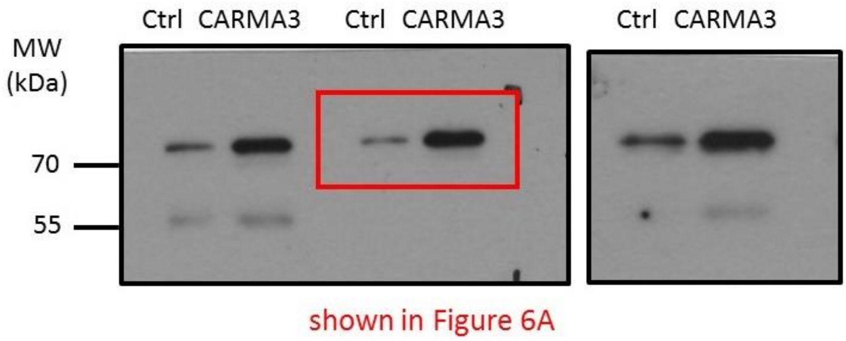

32. Figure S6A-a

Overexpression of CARMA3 by plasmid transfection
Cell: SW480 cell
Protein: Slug (30 kDa)

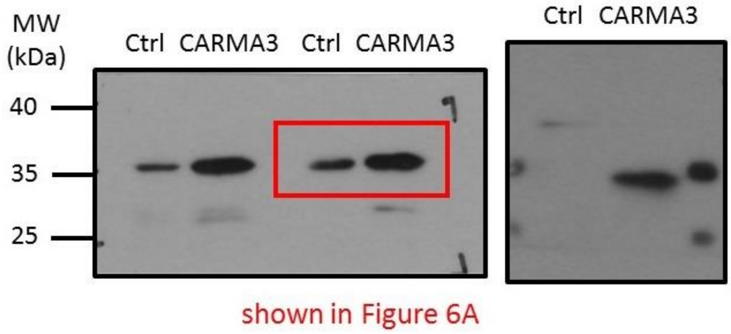

33. Figure S6A-a

Overexpression of CARMA3 by plasmid transfection
Cell: SW480 cell
Protein: α-Tubilin (55 kDa)

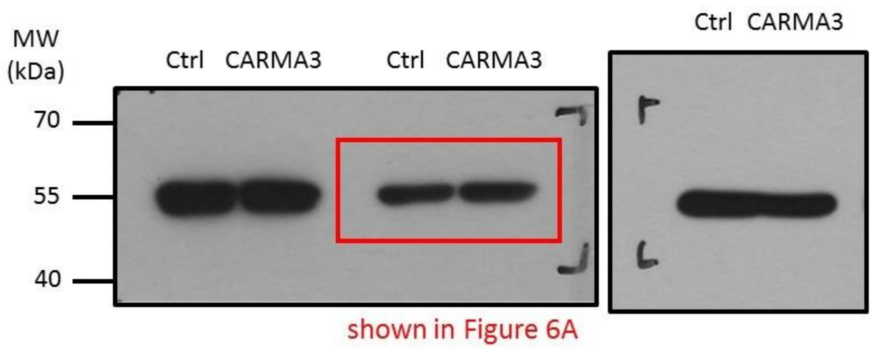

34. Figure S6A-b

Knockdown of CARMA3 by shRNA lentivirus infection
Cell: HCT116 cell
Protein: CARMA3 (116 kDa)

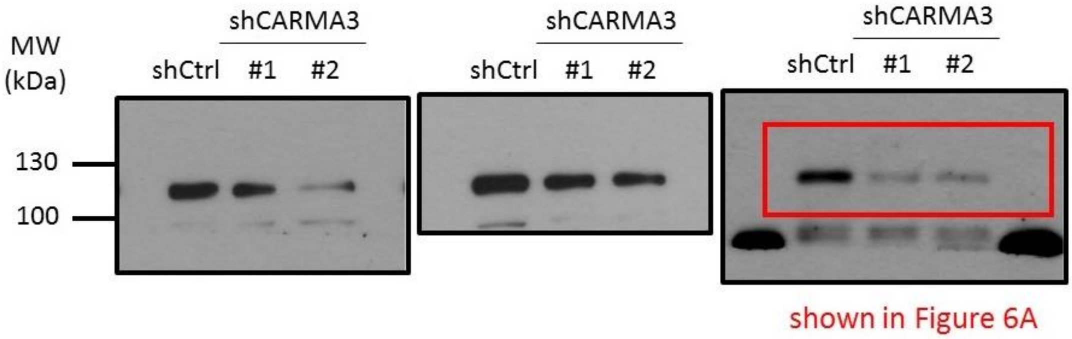

35. Figure S6A-b

Knockdown of CARMA3 by shRNA lentivirus infection
Cell: HCT116 cell
Protein: YAP (65-78 kDa)

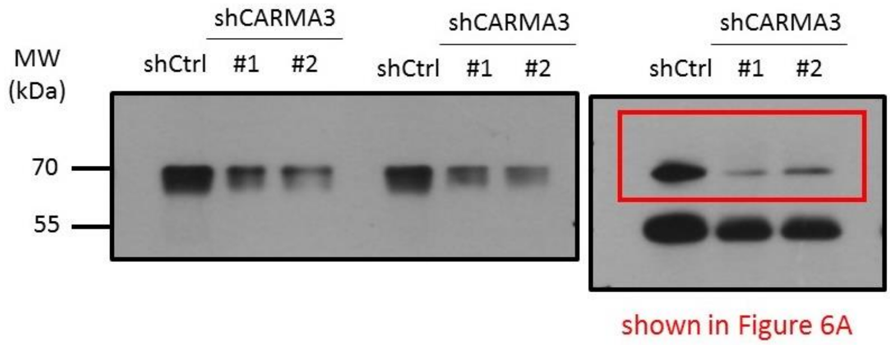

36. Figure S6A-b

Knockdown of CARMA3 by shRNA lentivirus infection
Cell: HCT116 cell
Protein: Slug (30 kDa)

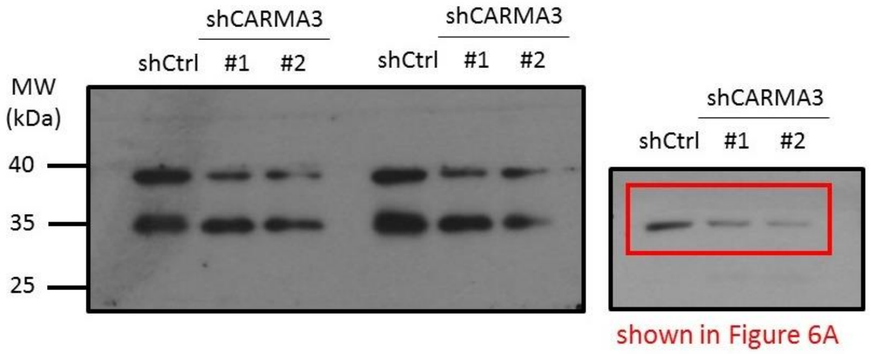

37. Figure S6A-b

Knockdown of CARMA3 by shRNA lentivirus infection
Cell: HCT116 cell
Protein: α-Tubilin (55 kDa)

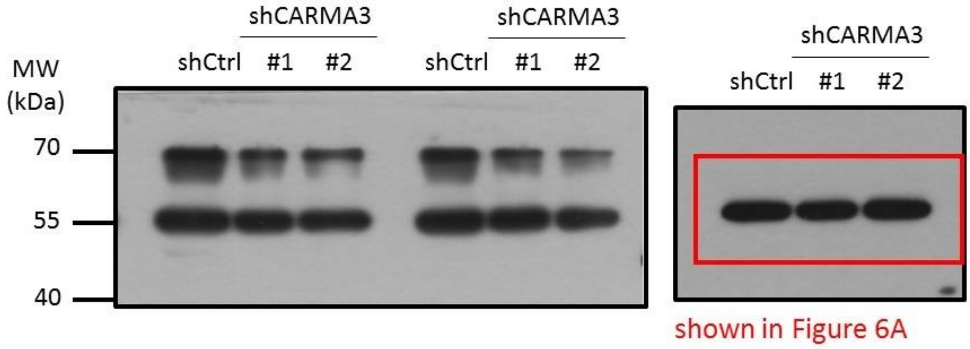

38. Figure S6C-a

Knockdown of YAP by shRNA lentivirus infection in CARMA3-overexpressed cells
Cell: SW480 cell
Protein: CARMA3 (116 kDa)

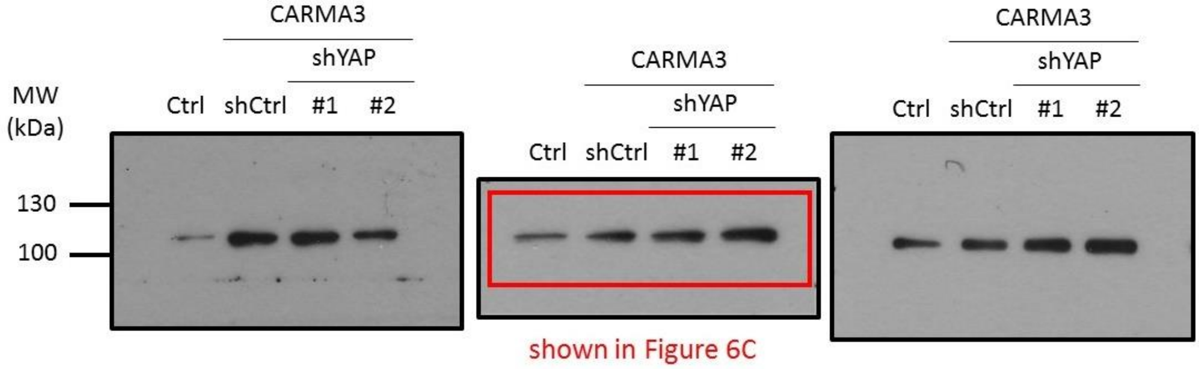

39. Figure S6C-a

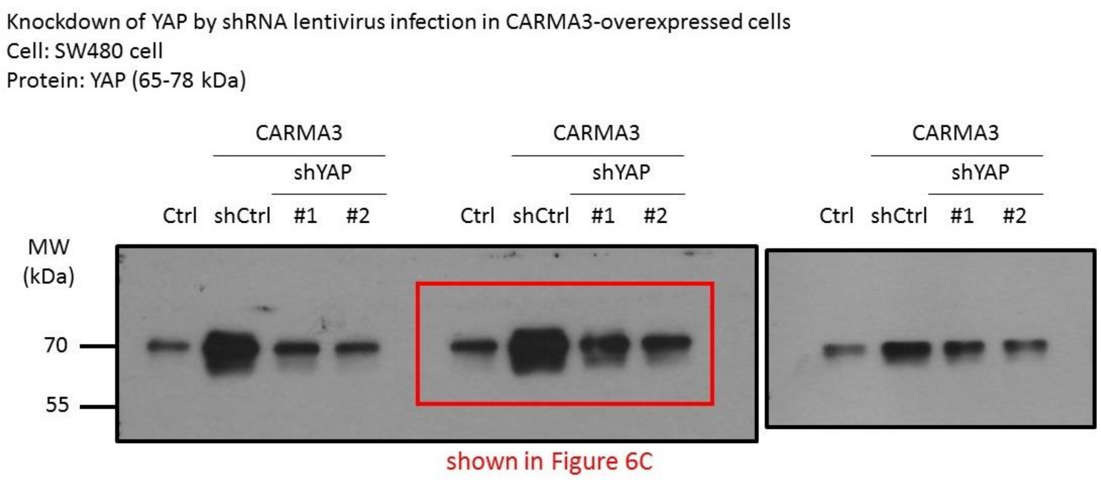

40. Figure S6C-a

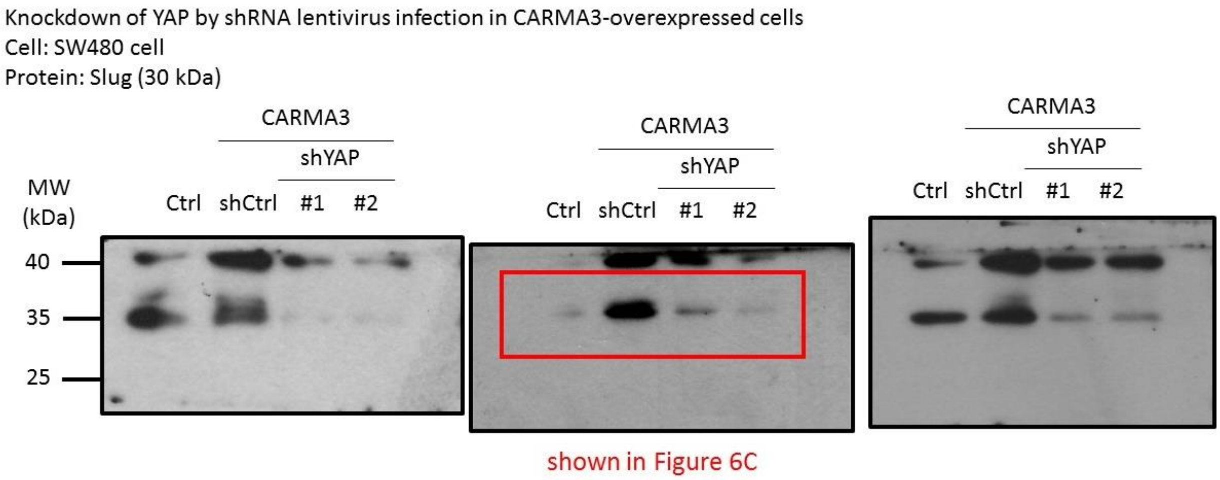

41. Figure S6C-a

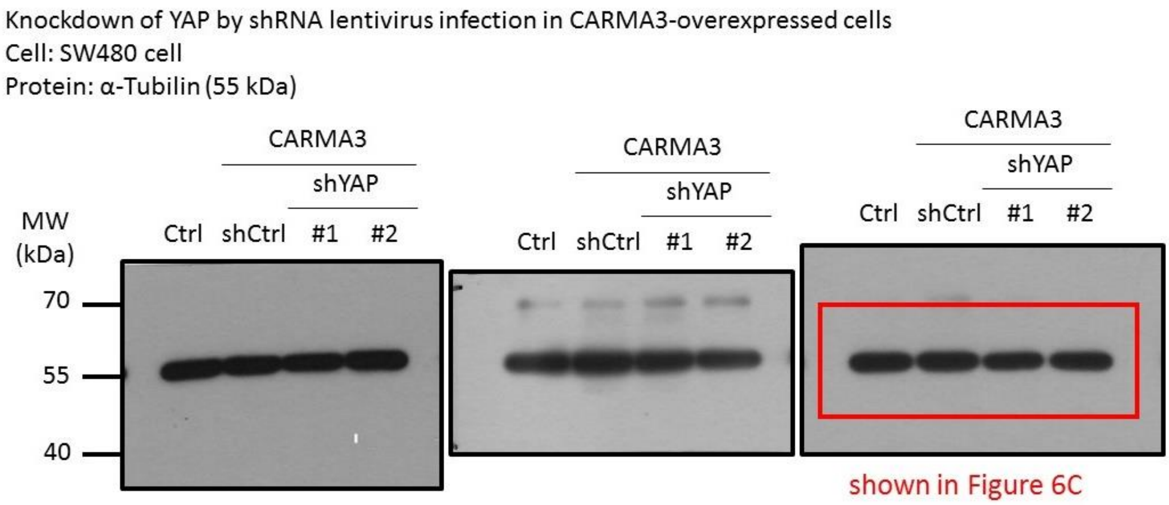

42. Figure S6C-b

Overexpression of YAP by plasmid transfection in CARMA3-silenced cells
Cell: HCT116 cell
Protein: CARMA3 (116 kDa)

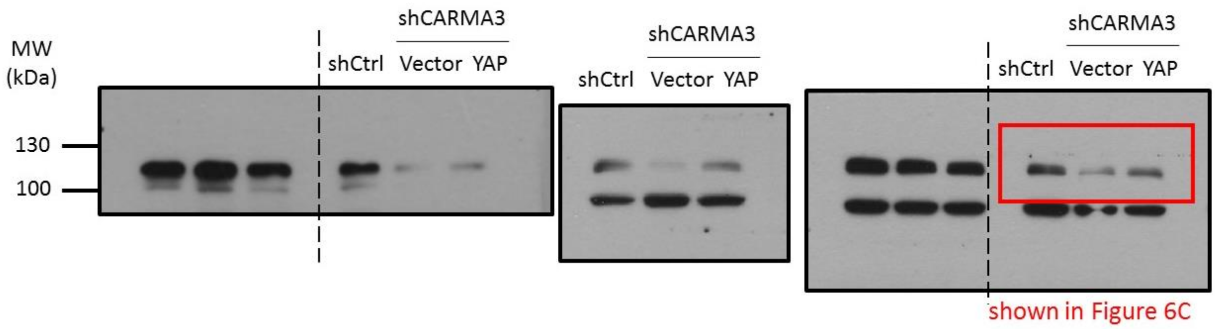

43. Figure S6C-b

Overexpression of YAP by plasmid transfection in CARMA3-silenced cells
Cell: HCT116 cell
Protein: YAP (65-78 kDa)

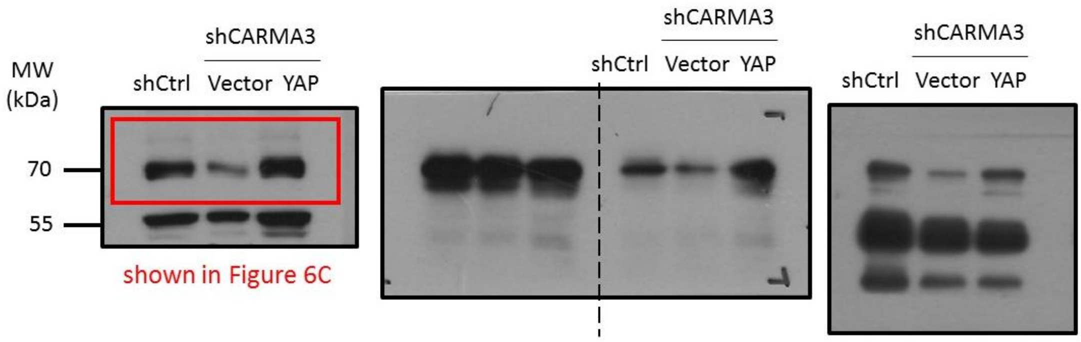

44. Figure S6C-b

Overexpression of YAP by plasmid transfection in CARMA3-silenced cells
Cell: HCT116 cell
Protein: Slug (30 kDa)

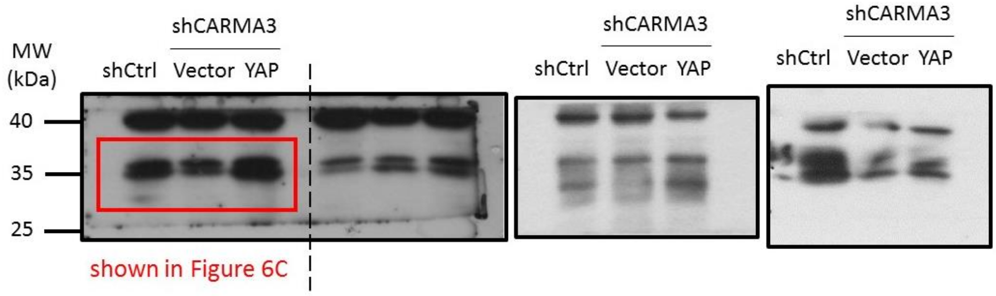

45. Figure S6C-b

Overexpression of YAP by plasmid transfection in CARMA3-silenced cells
Cell: HCT116 cell
Protein: α-Tubilin (55 kDa)

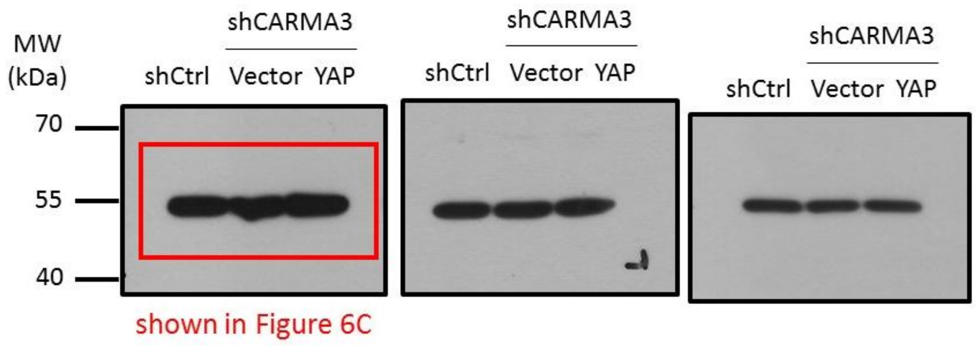

46. Figure S7A

Treatment with BAY or VP in CARMA3-overexpressed cells
Cell: SW480 cell
Protein: CARMA3 (116 kDa)

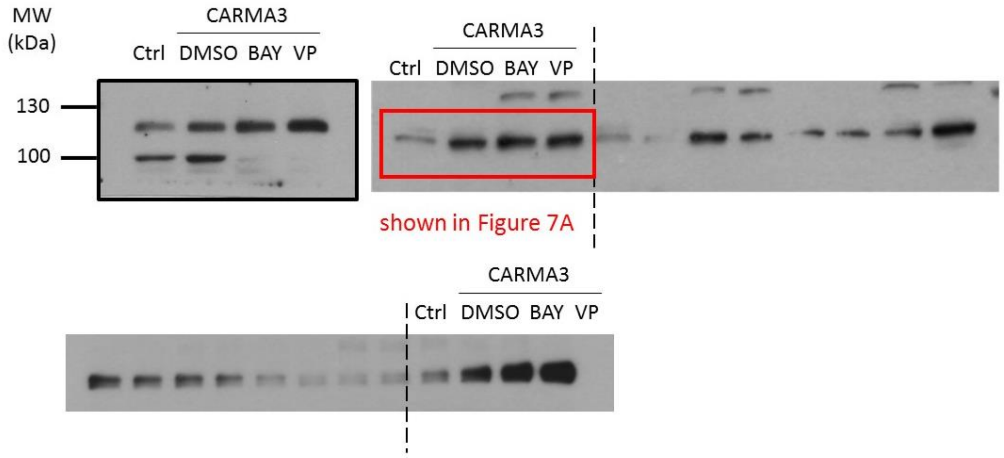

47. Figure S7A

Treatment with BAY or VP in CARMA3-overexpressed cells
Cell: SW480 cell
Protein: YAP (65-78 kDa)

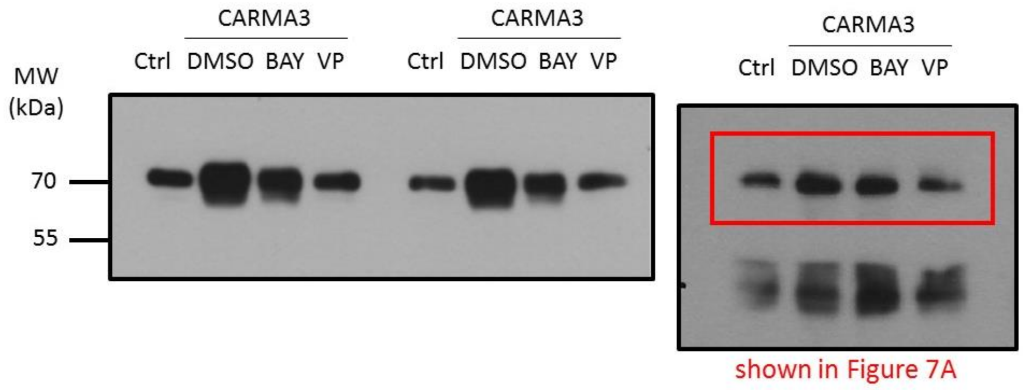

48. Figure S7A

Treatment with BAY or VP in CARMA3-overexpressed cells
Cell: SW480 cell
Protein: p-IκBα (40 kDa)

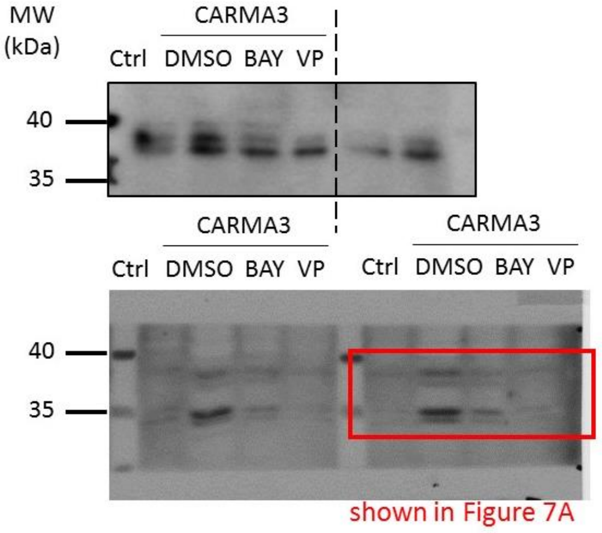

49. Figure S7A

Treatment with BAY or VP in CARMA3-overexpressed cells
Cell: SW480 cell
Protein: IκBα (35-41 kDa)

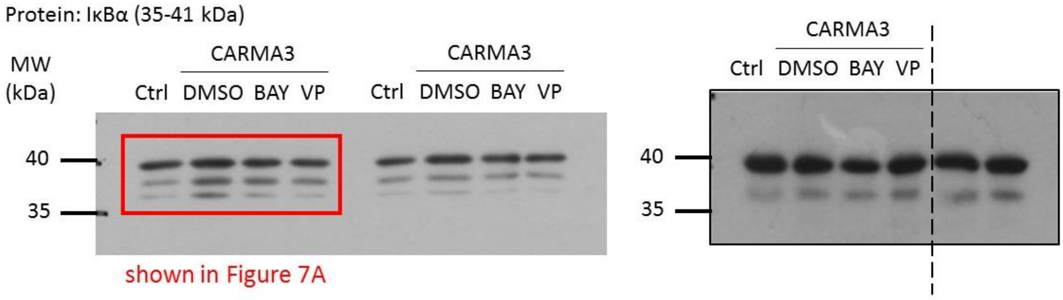

50. Figure S7A

Treatment with BAY or VP in CARMA3-overexpressed cells
Cell: SW480 cell
Protein: Slug (30 kDa)

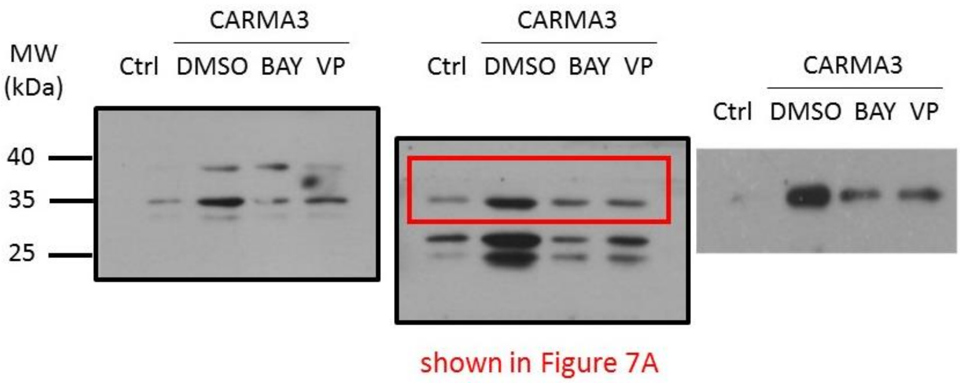

51. Figure S7A

Treatment with BAY or VP in CARMA3-overexpressed cells
Cell: SW480 cell
Protein: α-Tubilin (55 kDa)

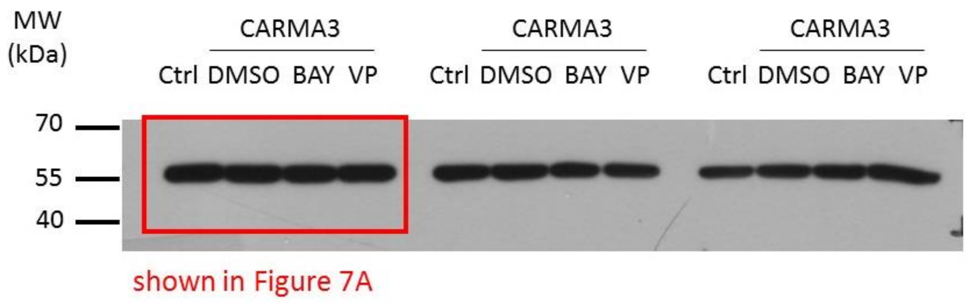

52. Figure S7C

Treatment with TNFα or VP in CARMA3-overexpressed cells
Cell: SW480 cell
Protein: CARMA3 (116 kDa)

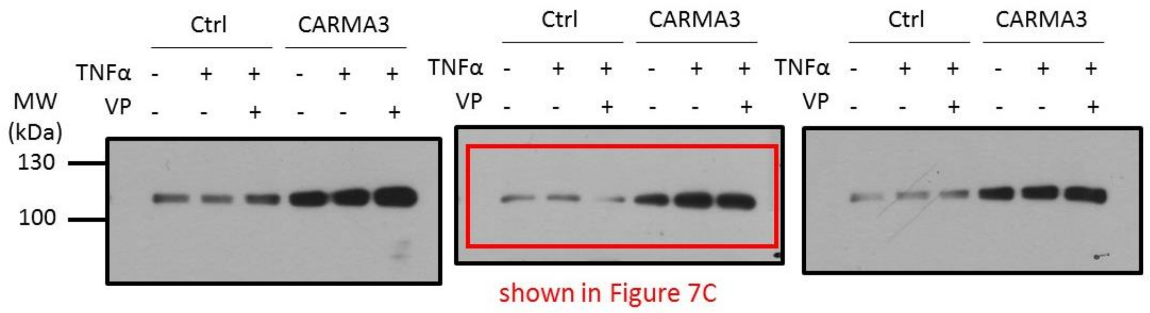

53. Figure S7C

Treatment with TNFα or VP in CARMA3-overexpressed cells
Cell: SW480 cell
Protein: YAP (65-78 kDa)

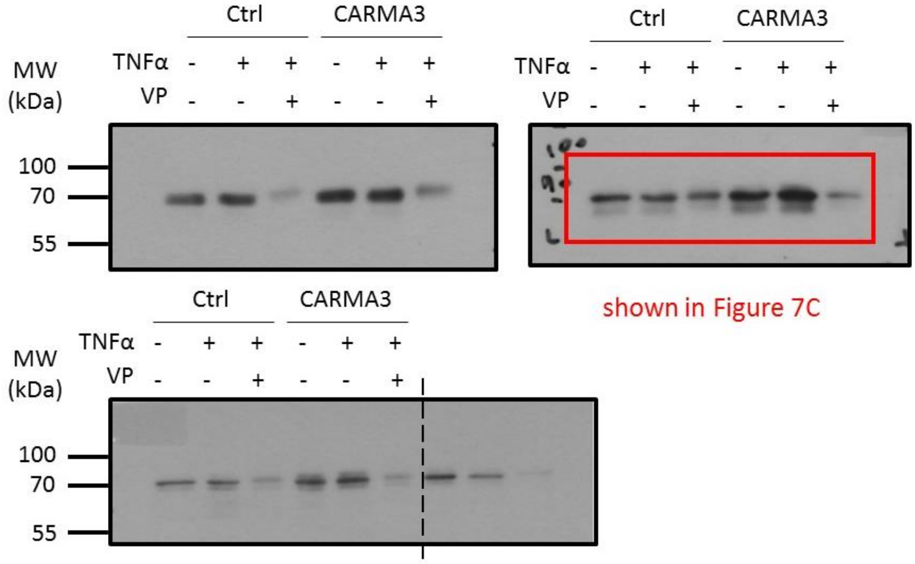

54. Figure S7C

Treatment with TNFα or VP in CARMA3-overexpressed cells
Cell: SW480 cell
Protein: p-IκBα (40 kDa)

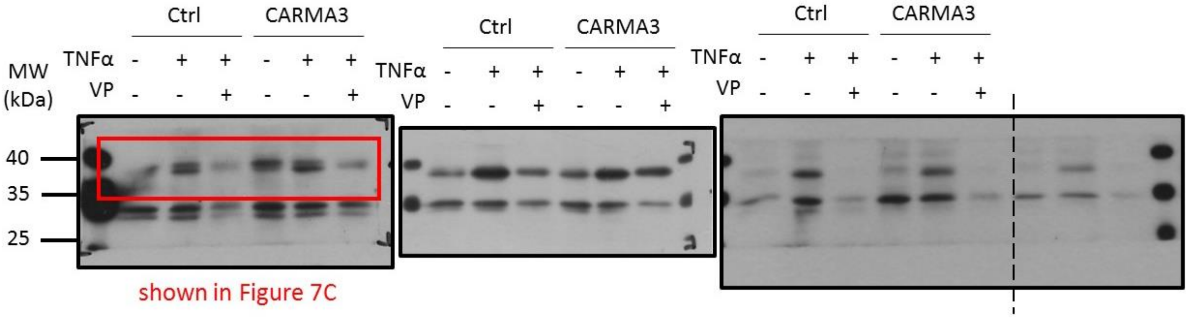

55. Figure S7C

Treatment with TNFα or VP in CARMA3-overexpressed cells
Cell: SW480 cell
Protein: IκBα (35-41 kDa)

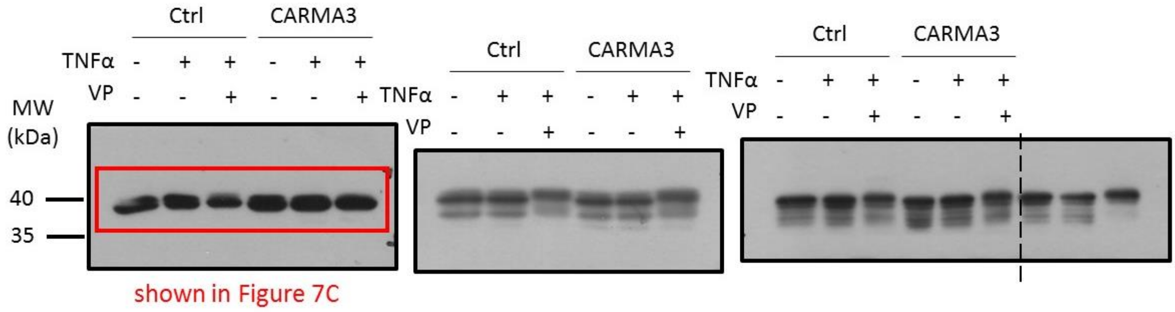

56. Figure S7C

Treatment with TNFα or VP in CARMA3-overexpressed cells
Cell: SW480 cell
Protein: Slug (30 kDa)

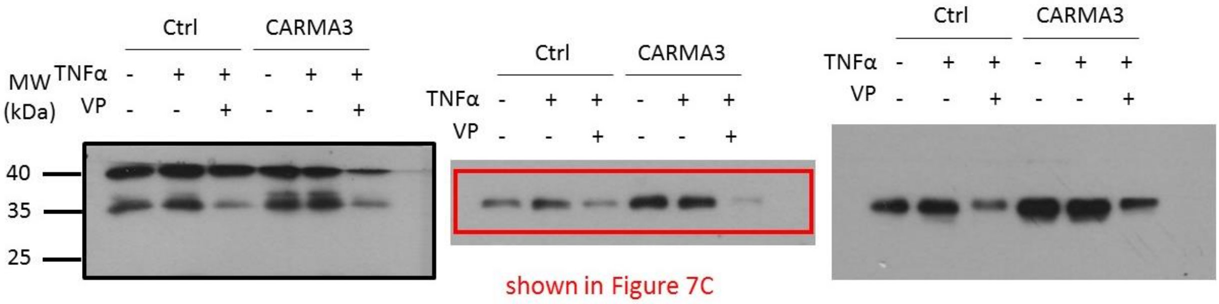

57. Figure S7C

Treatment with TNFα or VP in CARMA3-overexpressed cells
Cell: SW480 cell
Protein: α-Tubilin (55 kDa)

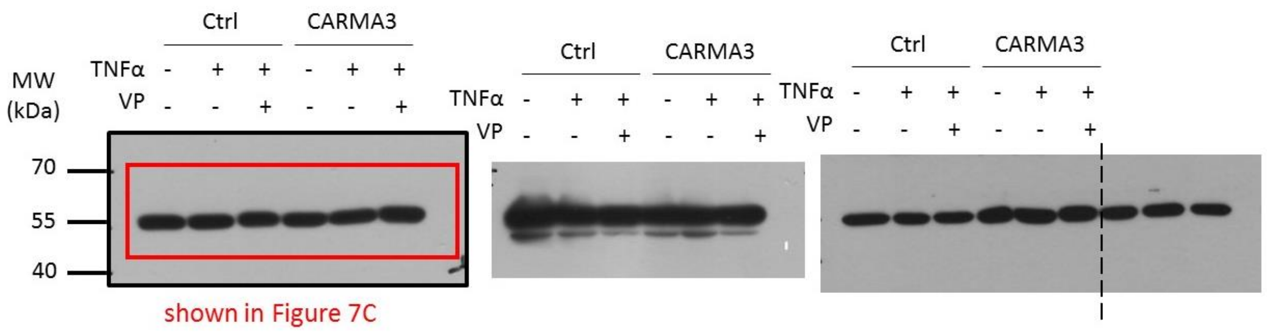

58. Figure S8A

Overexpression of CARMA3 by plasmid transfection in CARMA3-silenced cells
Cell: HCT116 cell
Protein: CARMA3 (116kDa)

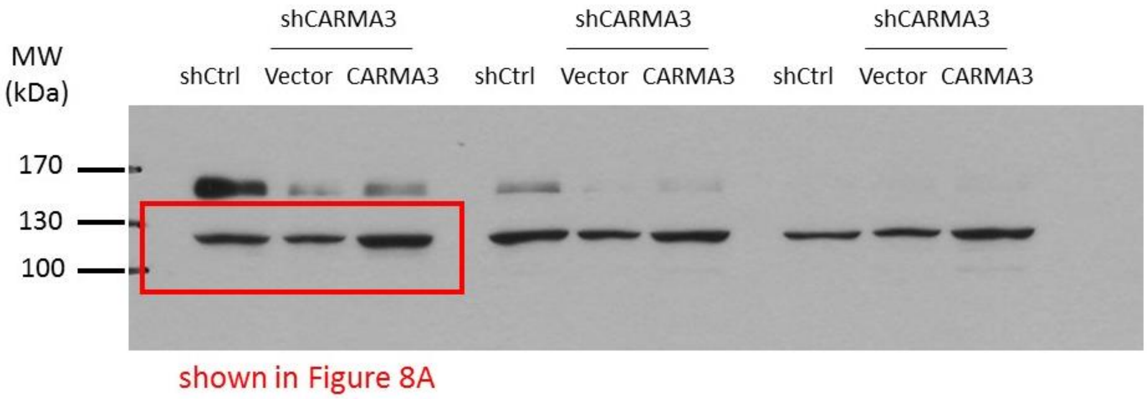

59. Figure S8A

Overexpression of CARMA3 by plasmid transfection in CARMA3-silenced cells
Cell: HCT116 cell
Protein: YAP (65-78 kDa)

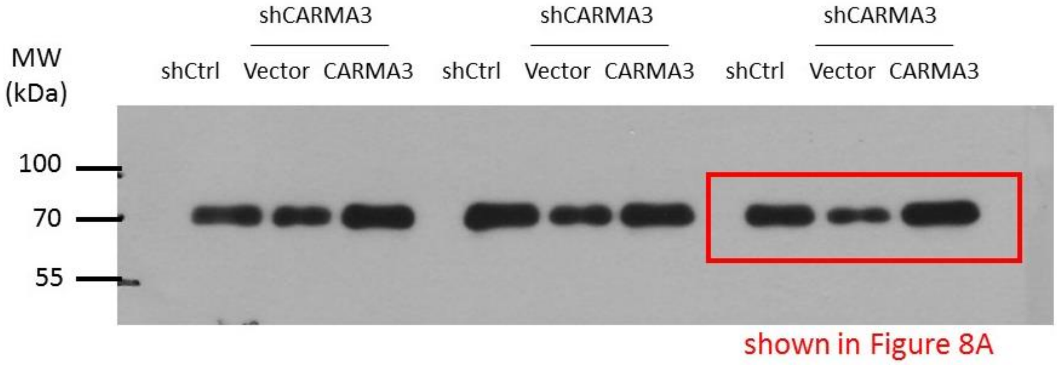

60. Figure S8A

Overexpression of CARMA3 by plasmid transfection in CARMA3-silenced cells
Cell: HCT116 cell
Protein: p-IκBα (40 kDa)

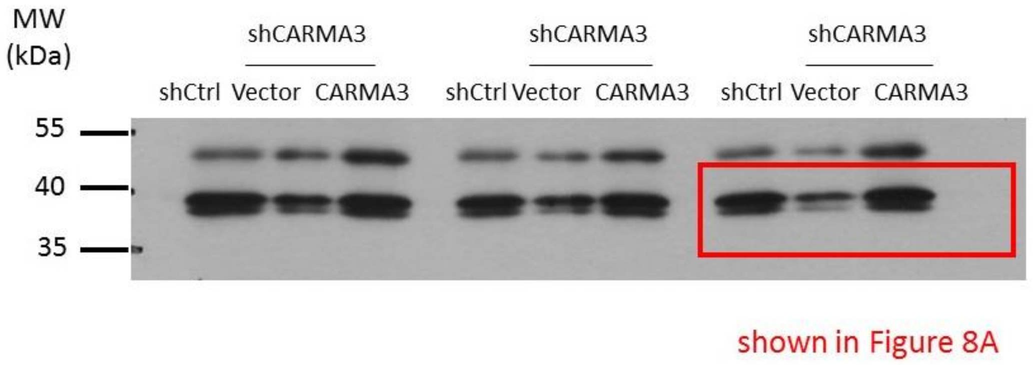

61. Figure S8A

Overexpression of CARMA3 by plasmid transfection in CARMA3-silenced cells
Cell: HCT116 cell
Protein: IκBα (35-41 kDa)

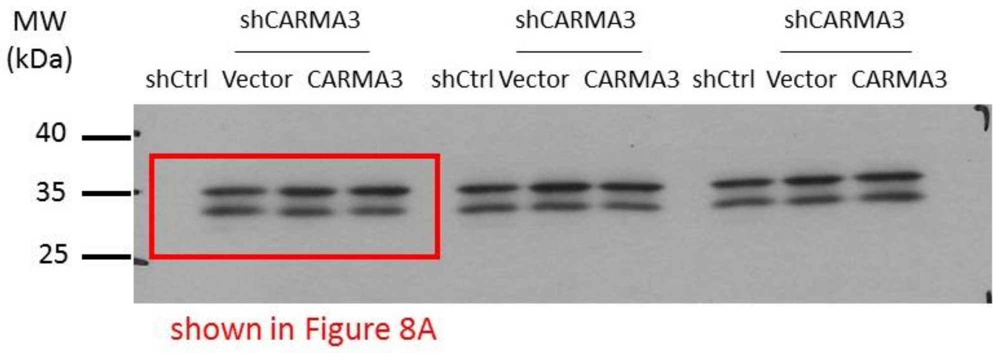

62. Figure S8A

Overexpression of CARMA3 by plasmid transfection in CARMA3-silenced cells
Cell: HCT116 cell
Protein: Slug (30 kDa)

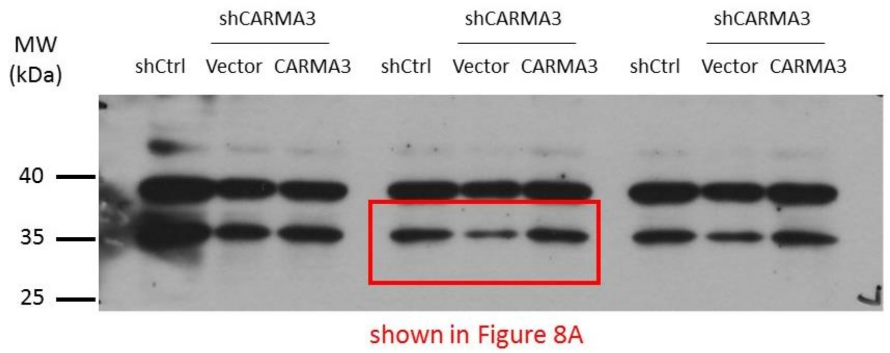

63. Figure S8A

Overexpression of CARMA3 by plasmid transfection in CARMA3-silenced cells
Cell: HCT116 cell
Protein: α-Tubilin (55 kDa)

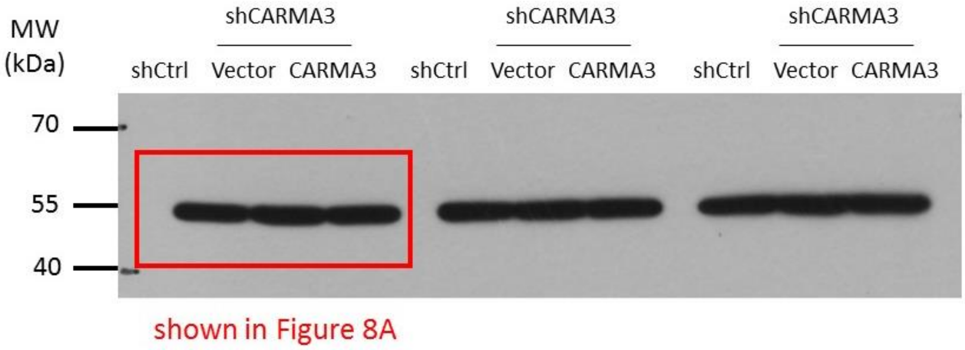

Supplement: Supplementary file 1 [file cancers-13-05946-s001.zip › cancers-1438805-supplementary.pdf]
